# Older patients with vertebral and pelvic fractures: Study protocol of a clinical cohort

**DOI:** 10.1371/journal.pone.0306727

**Published:** 2024-08-27

**Authors:** Patrick Roigk, Rebekka Leonhardt, Ulrich Lindemann, Bastian Abel, Gisela Büchele, Dietrich Rothenbacher, Jessica Koschate, Julia Schlotmann, Mohamed Elsayed, Tania Zieschang, Thea Laurentius, Cornelius Bollheimer, Clemens Becker, Kilian Rapp

**Affiliations:** 1 Department of Geriatrics, Robert-Bosch-Hospital, Stuttgart, Germany; 2 Institute of Epidemiology and Medical Biometry, Ulm University, Ulm, Germany; 3 Fakulty of Medicine and Health Sciences, Department for Health Services Research, University Oldenburg, Oldenburg, Germany; 4 Medical Department for Geriatric Medicine, University RWTH Aachen–Franziskus, Aachen, Germany; 5 Unit of Digital Geriatric Medicine, University Hospital, Heidelberg, Germany; National Trauma Research Institute, AUSTRALIA

## Abstract

**Background:**

Vertebral and pelvic fractures are associated with a significant burden of negative health and psychosocial outcomes. The number of vertebral and pelvic fractures is increasing in an aging society. Vertebral and pelvic fractures are increasingly significant injuries for individuals and society. However, few epidemiological studies have examined the clinical course of vertebral and pelvic fractures. This is the protocol for a study that observes patients who have been admitted to the hospital with an incident vertebral or pelvic fracture for a period of 12 months.

**Methods:**

The observational cohort study is conducted at three study sites in Germany. Patients affected by vertebral or pelvic fractures are recruited within the first few days of hospital admission. Data collection takes place at four-time points: baseline, before discharge, after 4 months, and after 12 months after admission to the hospital. Particular emphasis is laid on the assessment of the fall mechanisms, physical function, physical activity, life space, mobility, treatment approach, and quality of life. The hospital stay involves the collection of biomaterials (blood and urine).

**Discussion:**

The study aims to enhance understanding of the clinical progression and outcomes in patients with fractures in the vertebrae or pelvis.

## Introduction

A fragility fracture is a common occurrence in individuals over the age of 50, with approximately one in two women and one in four men experiencing it during their remaining lifetime [1]. With increasing age, fragility fractures occur for example at the forearm, the humerus, or the hip. Hip fractures are considered the primary fracture for osteoporotic fractures and are closely associated with mobility, disability, quality of life, and death. Therefore, numerous observational studies have been conducted, resulting in a substantial body of literature on the epidemiology of hip fractures [2].

Vertebral and pelvic fractures are two types of fragility fractures that commonly occur in older individuals. The incidence of these fractures is increasing in an aging society. Vertebral fractures in older individuals may result from falls or occur spontaneously due to low bone mineral density. Pelvic fractures are predominantly caused by falls.

The individual may experience chronic pain, functional impairments, disability, an increased risk of institutionalisation, and even death as a result [3–5]. Vertebral and pelvic fractures are significant injuries for both individuals and society. Therefore, it is important to consider their impact and treatment options objectively.

Epidemiologic studies on vertebral or pelvic fractures are limited, unlike hip fractures. There is limited data on the percentage of falls causing vertebral fractures and the correlation between fall mechanisms and fracture types due to lack of longitudinal study [6–8]. The EPOS study in Europe showed an overall incidence of 10.7/1000 person-years in women and 5.7/1000 person-years in men [9]. In addition, the long-term effects of pain, physical function, and physical activity are not well described in cases of vertebral and pelvic fractures. Both types of fractures can be treated either conservatively or surgically. The choice between the two treatment options depends on the type and classification of the fracture. However, less standardized parameters, such as the patient’s pain level, may also influence the therapeutic decision-making. Treatment types can vary between hospitals and countries due to the lack of standardization [5].

This article presents the protocol of a study cohort that examines patients with an incident vertebral or pelvic fracture over a 12-month period. Particular emphasis is laid on the assessment of the fall mechanism, physical function, physical activity, life space, mobility, and quality of life.

## Materials and methods

The study is a prospective cohort trial that follows patients with an incident vertebral or pelvic fracture for a period of 12 months. The study is implemented at three study sites (Robert-Bosch-Hospital, Stuttgart; University of Oldenburg, Oldenburg; University Hospital RWTH ‐ Franziskus Aachen) in Germany. The recruitment of patients started (first patient in) in Stuttgart on 30/01/2020; in Oldenburg on 22/04/2021 and Aachen on 17/06/2021. The recruitment of patients ended (last patient out) in Stuttgart on 30/04/2025; in Oldenburg on 31/07/2024 and Aachen on 30/04/2025. In Stuttgart, the cohort study was preceded by a cross-sectional study with an assessment only during the hospital stay from 07/2019–01/2020.

This study protocol is reported following the STROBE guideline.

### Participants

To be eligible for the study, patients must be 70 years or older and have been admitted to the hospital with a vertebral or pelvic fracture within the last 3 months. Patients with a vertebral fracture often undergo magnetic resonance tomography to determine the timing of the fracture and plan further treatment. This information, together with the patient’s clinical history, helps us estimate the age of the fracture. Table 1 provides an overview of all inclusion and exclusion criteria.

**Table 1:** Inclusion and exclusion criteria for study participation.

Inclusion criteria	Exclusion criteria
• age ≥70 years • admitted to the hospital with an incident acute or subacute, clinical apparent, vertebral or pelvic fracture • ability to understand the content of the study and to communicate a choice [10]	• old fracture (diagnosis >3 months ago) • pathological fracture due to a malignant disease • serious communication problems due to dysarthria/ aphasia /hearing inability to understand the German language • terminal illness • severe cognitive impairment (not able to understand the content of the study or to communicate a choice) • severe mental/psychiatric disease • no informed consent due to impaired cognition or lack of informed consent by a legal representative • place of living more than one hour away from the study site by public transport • inability to walk independently for a short distance with or without walking aids before the index fracture

### Ethics approval and consent to participate

**Fig 1:**
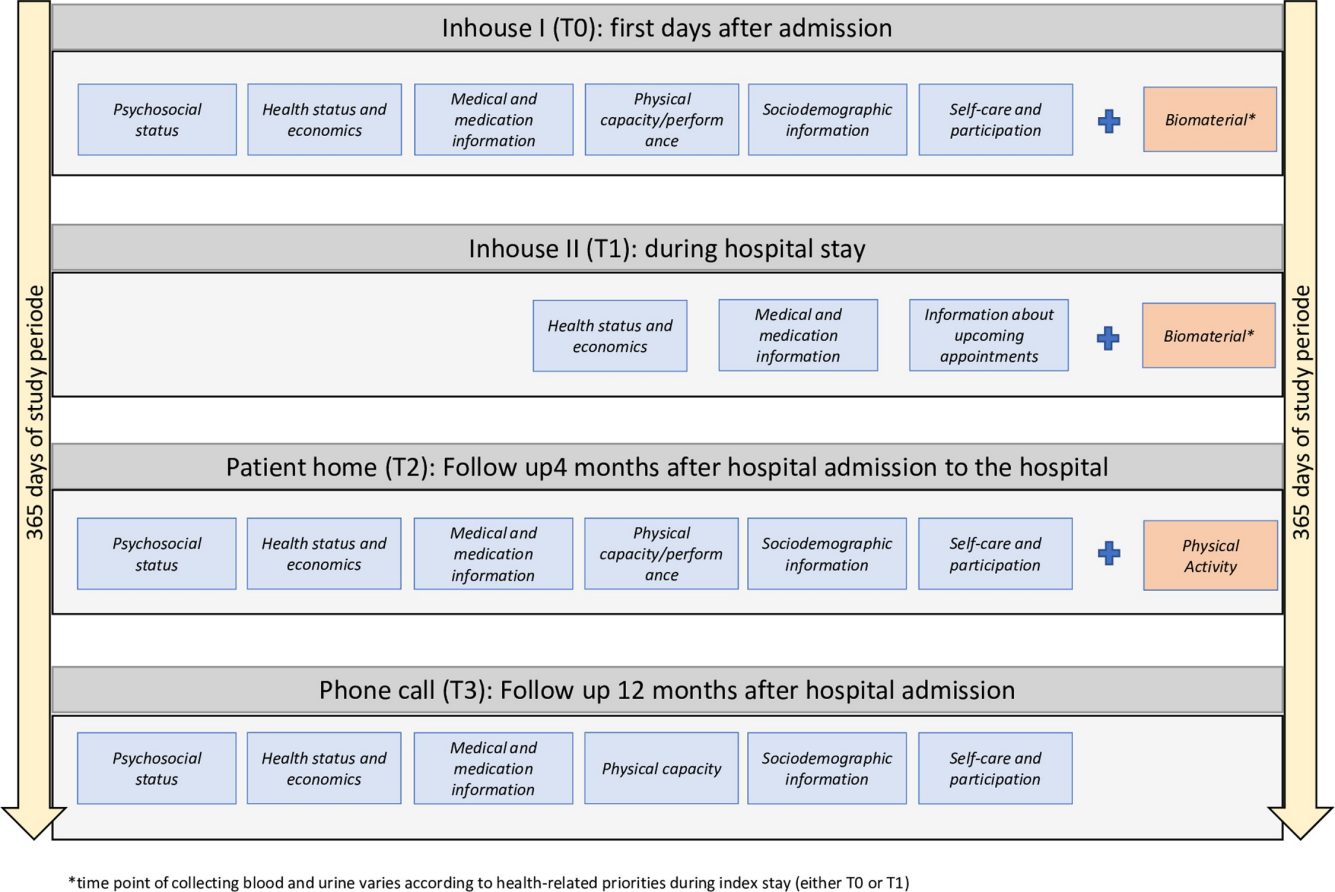
Process of data collection and time points.

The study has been approved by the local ethics committees at each study site (Stuttgart: Ethics Committee of the Medical Faculty of the Eberhard-Karls-University and the University Hospital Tübingen [approval 879/2019BO2]), Aachen: Ethics Committee of the Medical Faculty RWTH Aachen [approval 21–043], and Oldenburg: Ethics Committee of the Medical Faculty of the University Oldenburg [approval 2020–136]). All measurements in this study involving human participants are following the latest revision (2013 in Fortaleza, Brasilia) of the 1964 Helsinki Declaration. All patients are provided with comprehensive verbal and written information about the study. Following the initial T0 routine assessment, patients are requested to provide informed consent (refer to Fig 1). Patients have the right to withdraw their informed consent at any time, without providing a reason.

**Trial registration:** German Clinical Trials Register, DRKS00023333. Registered on January 13, 2021 (https://drks.de/search/en/trial/DRKS00023333).

### Study protocol

#### Process of data collection and time points

The study collected data at four time points (T0-T1). These included two during the index fracture hospital stay, one at the patient’s home 120 days (4 months) after hospital admission (T2), and one via telephone 365 days (12 months) after hospital admission (T3) (refer to S1 Fig SPIRIT schedule_FriDA).

The dimensions addressed at different time points of data collection include sociodemographic information, medical information, psychosocial status, self-care and participation, health status and economics, physical capacity, physical activity, and biomaterials. Table 2 provides information on variables, assessments, source of information, and collection time. The study staff received comprehensive training to ensure standardized assessments.

**Table 2:** Dimensions, variables, source of information/assessment instrument, and time of collection.

Dimension	Variable	Source of information/ assessment instrument	Timepoint			
			T0	T1	T2	T3
*Socio-demographic information*	Date of birth, gender	Patient interview and hospital information system	X			
	Education (school years/ professional training years)	Patient interview and hospital information system	X			
	Living situation and living conditions	Patient interview and hospital information system	X^2^		X	X
	Degree of care	According to the German classification of care dependency (the Social Code (SGB XI))	X		X	X
*Medical information*	Fall history in the past 12 months before the index fracture	Self-developed questionnaire	X^2^			
	Fall or non-fall-related mechanism behind the index fracture	Self-developed questionnaire	X			
	Admission- / discharge date	Hospital information system	X	X		
	Treatment procedure	Patient interview and hospital information system		X	X^2^	X^2^
	Fracture diagnoses	International Classification of Disease (ICD 10)	X		X^2^	X^2^
	Height/ weight / Body mass index (BMI)	Hospital information system	X			
	Charlson comorbidity index (CCI)	Hospital information system	X			
	Pain medication (prescribed medication or medication taken within the last 7 days)	Self-developed questionnaire along the WHO Analgesic Ladder			X	X
	Osteoporosis medication	Self-developed questionnaire	X^2^		X	X
	Risk of fracture	Fracture Risk Assessment Tool (FRAX) [11]	X			
	T-Score	Dual X-ray Absorptiometry (DXA)	X		X	X
	Falls after discharge	Fall calendar over 12 months		X	X	X
*Psychosocial status*	Social support	Oslo-3-Items-Social-Support Scale« (Oslo-3) [12, 13]			X^2^	X^2^
	Emotion	Depression in older age (DIA-S) [14]	X		X	X
	Cognition	Blessed Orientation-Memory-Concentration-Test (BOMCT) [15]	X			
	Fall associated self-efficacy	The Falls Efficacy Scale International (FES-I short form) [16]			X	X
	Fear of falling	1-item question	X			
	Beliefs and motivation regarding physical exercise	Self-developed questionnaire			X	X
	Time of sedentary and activity (in combination with the sensor)	Self-developed questionnaire			X	
*Self-care and participation*	Life space mobility	Alabama Life-Space Assessment (LSA) [17]	X^2^		X^2^	X^2^
		Nursing home life space diameter [18]	X^2^		X^2^	X^2^
	Self-care ability	Barthel Index [19]	X			
*Health Status and Economics*	Quality of life	EQ-5D-3L [20]	X		X	X
	Osteoporosis-related quality of life	Quality of Life Questionnaire of the European Foundation for Osteoporosis: QUALEFFO-41 (in T0 only subscale ADL, iADL) [21]	X		X	X
	Fracture related pain	Self-developed questionnaire	X		X	X
	Pain intensity (during activity and inactivity)	Numeric rating scale (NRS) [22]	X	X	X	X
	Frequency of pain 6 months before index fracture	Self-developed rating scale	X^2^			
	Body composition	Bioelectrical Impedance Analysis ‐ BIA (AKERN BIA 101 N/H, SMT medical GmbH & Co. KG, Würzburg, D)	X			
	Use of professional or non-professional health service	Self-developed questionnaire	X^2^		X^2^	X^2^
	Current place of being (at home / short-term care/nursing home/hospital/rehabilitation	Self-developed questionnaire			X^2^	X^2^
*Physical capacity/ performance*	Physical performance	de Morton Mobility Index (DEMMI) [23]	X		X	
		Short Physical Performance Battery (SPPB) [24]			X	
	Walking ability	New mobility score (NMS) [25, 26]	X^2^		X	X
		Self-developed questionnaire	X^2^		X	X
	Handgrip strength	Dynamometer (Jamar, Saehan Corporation, South Korea)	X		X	
*Physical activity*	Sensor-based measurement of physical activity over 7 days	ActivPAL4 micro (PAL Technologies, Glasgow, UK)			X	
*Biomaterial*	Blood and urine sample	EDTA Full Blood 2,7 ml	X^3^	X^3^		
		EDTA Plasma 7,5 ml	X^3^	X^3^		
		Serum 4,7 ml	X^3^	X^3^		
		Urine Sample 10–12 ml	X^3^	X^3^		

^2^ retrospective assessment

^3^-time point of collecting blood and urine varies according to health-related priorities during index stay (either T0 or T1)

T0 = 2–10 days after admission; assessment on the surgical ward

T1 = 1–3 days before discharge; assessment on the surgical ward

T2 = 120 days after admission to the surgical ward; assessment in the patient´s home

T3 = 365 days after admission to the surgical ward; assessment via telephone

### T0: After admission/ during the hospital stay

- All patients admitted to the hospital for surgical reasons undergo screening for vertebral or pelvic fractures using the digital hospital information system (KIS). The KIS includes all medical information pertaining to the patient’s hospitalization.
- If a positive screening occurs, hospital or study staff will approach patients within the first few days of admission to carry out the initial T0 Assessment. The primary objective of this T0 assessment is to determine whether the fracture was caused by a fall or other factors. Additionally, standard assessments of mobility, emotion, and cognition will be conducted.
- After obtaining informed consent from patients, biomaterials such as blood and urine are collected. The biomaterials are prepared and aliquoted for freezing at -20 degrees Celsius (blood) and -80 degrees Celsius (urine) before being stored in a bio database for subsequent analysis. The protocol for preparing, aliquoting, and freezing biomaterials is described separately. This protocol was developed by the Dr. Margarete Fischer-Bosch-Institute of Clinical Pharmacology in Stuttgart.

### T1: Few days before discharge of the index hospital stay

- A few days prior to discharge, participants are given a ’fall calendar’.
- Participants were instructed by study staff to document all falls that occurred between hospital discharge and T3, which is 12 months after the initial hospital admission.

- In addition, the discharge location, current pain status, and treatment type for the index fracture are documented.

### T2: 4 months after hospital admission (home visit)

- Prior to T2, participants receive a phone call to schedule a home visit.
- The focus of T2 is to repeat the assessments performed during the index stay, including mobility, emotion, cognition, and any additional fractures or medical interventions since discharge.
- In addition, a sensor is attached to the participant’s thigh to assess their physical activity over a period of 7 days.

### T3: 12 months after hospital admission (phone call)

- T3 is carried out by telephone (see Table 2).
- At T3, a range of variables including socio-demographic information (such as living situation), medical information (such as incident fractures), psychosocial status (such as fall-associated self-efficacy), participation (such as life space assessment), health status (such as pain intensity) and physical performance (such as walking ability) are updated.

#### Consent for participation

Informed consent is a prerequisite for study participation after receiving both verbal and written information. Furthermore, all participants were given the option to decline participation in certain assessments, such as providing biomaterial or wearing a physical activity sensor.

### Study size

The study began at each study site after receiving approval from the respective ethics committees. The study is an open cohort with ongoing recruitment. The expected number of cases is between 30 and 80 patients per year at each study site.

### Statistics

The evaluation will adhere to the standards of epidemiological analyses in observational studies. This includes descriptive and association measures, as well as the use of multivariate methods to minimize the risk of bias and confounding.

Multivariate methods will be used to analyse the predictors of change in physical capacity, psychological status, and social participation among patients with vertebral and pelvic fractures over a period of 12 months.

## Discussion

We hereby present the study protocol and the objectives of a prospective cohort study on patients with incident vertebral or pelvic fractures. Numerous epidemiological studies have been conducted and published on the two types of fractures, using either register or routine health claims data. For vertebral fractures, register based data are available for various countries including Ireland [27], Sweden [28], Netherlands [29], and Germany [30]. The studies mentioned above have mainly focused on the prevalence or incidence of vertebral fractures, their risk factors, treatment characteristics, comorbidities, and/or mortality rates. We found only one prospective study from UK [27, 31, 32] that examined data from patients who were prospectively recruited in the hospital and had a confirmed or suspected vertebral fracture [31, 32]. This study presents data collected from patients during their hospitalization, covering demographics, co-pathology, frailty status, cognition, mood, fall history, pain, and physical functioning. A follow-up was conducted six months after discharge. Data collection included pain, physical functioning, residential status, mortality, and quality of life [32]. Additional studies have examined the epidemiology of pelvic fractures. These studies have used patient data from the USA [33], Belgium [34], Austria [35], and Germany [36, 37] to report on the incidence rate, type of fracture, and mortality rate in different cohorts. These studies also based on register data. Only two prospective epidemiological studies have been identified so far: one from India [38] and another from Germany [39]. Hack et al. conducted a study concerning the long-term outcome of activities of daily living (ADL) and self-reported health status (HS) in patients with pelvic fractures [39]. The observation period included radiological examination at six weeks and telephone follow-up at 6 and 12 months after the fracture occurred.

In summarising the evidence on the epidemiology of vertebral and pelvic fractures in older people, it is important to note that there is limited information available regarding their incidence, prevalence, and mortality. However, most studies were based on register or routine data, which often lack information about disabilities in daily activities, functional outcomes such as physical activity and capacity, subjective parameters like pain or quality of life, or geriatric syndromes such as sarcopenia or falls.

The aim of our study is to enhance understanding of the clinical course of patients following a vertebral or pelvic fracture incident. Both fracture types can be treated either conservatively or surgically. The study specifically focuses on subsequent dimensions and assessments, and research questions:

- Vertebral fractures can occur spontaneously or as a result of a traumatic event. Data on the prevalence of one of the two causes of a fracture is limited [40]. Therefore, it is important to provide a detailed history of a potential fall and its health-related outcomes. What percentage of fractures (clinically apparent and hospitalized) attributed to falls?
- Physical activity: both fracture types have an important impact on physical activity. However, there is currently limited knowledge regarding physical activity levels after discharge. To address this gap in knowledge, body-worn sensors are used to measure physical activity over a 7-day period, 4 months after discharge.
- Physical capacity: Individuals with vertebral or pelvic fractures often experience a significant reduction in motor function. Hence, alterations in the parameters pertaining to physical capacity may be valuable indicators for the clinical trajectory within the subsequent examinations. Utilized tools in geriatric assessments include gait speed, 5 chair rise, and composite scores such as the de Morton Mobility Index [41].
- Pain is typically the predominant symptom in both types of fractures and is often strongly associated with high or low physical activity. Hence, pain assessment is consequently conducted at baseline and follow-up.
- Life space mobility is a concept that is determined by physical capacity, physical activity, and pain [42]. Furthermore, life space may serve as a valuable tool to observe the clinical progression in patients with vertebral or pelvic fractures, as it may be a surrogate of social participation.
- Falls are a common cause of vertebral and pelvic fractures, accounting for a considerable proportion of cases. Subsequently, secondary fractures following an index fracture (imminent fractures) are common [43, 44]. Therefore, in our study, falls are assessed prospectively for 12 months after discharge.
- The ability to perform the instrumental activities of daily living (I-ADL) is particularly at risk in frail elderly individuals who have experienced a fracture, and this can subsequently impact their quality of life (QoL) [45]. At baseline and during follow-up, the assessment of I-ADLs, degree of disability, and QoL takes place.
- The Biobanking has been established. Patients provide blood and urine specimens, which are processed according to the established protocol within a designated timeframe. Bone metabolism with biomarkers like parathyroid hormone (PTH), Tartrate-resistant acid phosphatase 5b (TRACP 5b), N-terminal propeptide of type I procollagen (PINP), osteocalcin or sclerostin will be examples of biomarkers that are of interest.

The study design allows a variety of scientific questions. Examples include determining the percentage of vertebral fractures caused by falls among clinically apparent and hospitalized cases, or comparing fracture localization and classification between fractures caused by falls and those that occurred spontaneously. Additionally, an analysis will be carried out on the factors that affect physical function, physical activity, falls, or participation following a vertebral or pelvic fracture. An additional scientific inquiry is the clinical impact of a conservative or surgical approach on the outcomes mentioned above in patients with vertebral or pelvic fractures.

The study has several strengths, including a detailed characterization of the patients, a prospective design, and the participation of three clinical recruiting sites across Germany (in the South, Middle, and North).

The study design has also limitations. The baseline data is partly derived from clinical routine and partly assessed by study nurses. The reliability of the information may be reduced due to the rate of participation being less than 100%, which could potentially lead to the presence of selection bias. Patients who experience fractures frequently develop cognitive impairment and delirium up to 57,6% pre-operatively and 41.7% post-surgery [46]. Therefore, we reduce the risk of information bias, it is helpful to include and combine additional information such as data from the emergency department or testimonies provided by proxies, particularly in the detailed assessment of the fracture-related fall event. Patient recruitment is restricted to a single country, only hospitals and three study centres. Variations in treatment types, such as surgical indications, length of hospital stay, and the extent of orthogeriatric co-management or subacute rehabilitation, may differ between institutions and countries which influence the mortality rate, length of stay or 30-day readmission rate [47]. These differences may affect the generalisability of the results.

## Conclusions

In summary, this is the study protocol for a clinical cohort of patients with incident vertebral or pelvic fractures. The study aims to enhance our understanding of the clinical course and outcomes in this population of older persons and often frail patients.

Study protocol: Vs 1

## Supporting information

S1 Fig SPIRIT schedule_FriDA. (DOCX)

S1 File Supporting Information_Study protocol Engl. (PDF)

S2 File Supporting Information_Study protocol German. (PDF)

## Abbreviations

PR: Patrick Roigk
RL: Rebekka Leonhardt
UL: Ulrich Lindemann
BA: Bastian Abel
GB: Gisela Büchele
DR: Dietrich Rothenbacher
JK: Jessica Koschate
JS: Julia Schlotmann
ME: Mohamed Elsayed
TZ: Tania Zieschang
TL: Thea Laurentius
CoB: Cornelius Bollheimer
CB: Clemens Becker
KR: Kilian Rapp
BMI: Body mass index
ICD: international classification of disease
CCI: Charleston comorbidity index
WHO: World Health Organization
FRAX: Fracture Risk Assessment Tool
DXA: Dual X-ray Absorptiometry
DIA-S: Depression in older age
BOMCT: Blessed Orientation-Memory-Concentration-Test
FES-I: The Falls Efficacy Scale International
LSA: Life Space Assessment
EQ-5D-3L: European Quality of Life 5 Dimensions 3 Level Version
QUALEFFO-41: Quality of Life Questionnaire of the European Foundation for Osteoporosis
iADL: Instrumantal Activity of Daily Living
BIA: Bioelectrical Impedance Analysis
DEMMI: de Morton Mobility Index
SPPB: Short Physical Performance Battery
NMS: New mobility score
