# Supplementary material for: Older patients with vertebral and pelvic fractures: Study protocol of a clinical cohort
Source: PLoS One. 2024 Aug 27;19(8):e0306727. doi: 10.1371/journal.pone.0306727 (PMC11349230; doi:10.1371/journal.pone.0306727)
Supplement: S1 Fig — (DOCX) [file pone.0306727.s001.docx]

| **Dimension** | **Variable** | **Source of information/ assessment instrument** | **Timepoint** | | | | |
| --- | --- | --- | --- | --- | --- | --- | --- |
|  |  |  | **T0** | **T1** | **T2** | **T3** |  |
| *Socio-demographic information* | Date of birth, gender | Patient interview and hospital information system | X |  |  |  |  |
|  | Education (school years/ professional training years) | Patient interview and hospital information system | X |  |  |  |  |
|  | Living situation and living conditions | Patient interview and hospital information system | X^2^ |  | X | X |  |
|  | Degree of care | According to the German classification of care dependency (the Social Code (SGB XI)) | X |  | X | X |  |
| *Medical information* | Fall history in the past 12 months before the index fracture | Self-developed questionnaire | X^2^ |  |  |  |  |
|  | Fall or non-fall-related mechanism behind the index fracture | Self-developed questionnaire | X |  |  |  |  |
|  | Admission- / discharge date | Hospital information system | X | X |  |  |  |
|  | Treatment procedure | Patient interview and hospital information system |  | X | X^2^ | X^2^ |  |
|  | Fracture diagnoses | International Classification of Disease (ICD 10) | X |  | X^2^ | X^2^ |  |
|  | Height/ weight / Body mass index (BMI) | Hospital information system | X |  |  |  |  |
|  | Charlson comorbidity index (CCI) | Hospital information system | X |  |  |  |  |
|  | Pain medication (prescribed medication or medication taken within the last 7 days) | Self-developed questionnaire along the WHO Analgesic Ladder |  |  | X | X |  |
|  | Osteoporosis medication | Self-developed questionnaire | X^2^ |  | X | X |  |
|  | Risk of fracture | Fracture Risk Assessment Tool (FRAX)^9^ | X |  |  |  |  |
|  | T-Score | Dual X-ray Absorptiometry (DXA) | X |  | X | X |  |
|  | Falls after discharge | Fall calendar over 12 months |  | X | X | X |  |
| *Psychosocial status* | Social support | Oslo-3-Items-Social-Support Scale« (Oslo-3)^10,11,11^ |  |  | X^2^ | X^2^ |  |
|  | Emotion | Depression in older age (DIA-S)^12^ | X |  | X | X |  |
|  | Cognition | Blessed Orientation-Memory-Concentration-Test (BOMCT)^13^ | X |  |  |  |  |
|  | Fall associated self-efficacy | The Falls Efficacy Scale International (FES-I short form)^14^ |  |  | X | X |  |
|  | Fear of falling | 1-item question | X |  |  |  |  |
|  | Beliefs and motivation regarding physical exercise | Self-developed questionnaire |  |  | X | X |  |
|  | Time of sedentary and activity (in combination with the sensor) | Self-developed questionnaire |  |  | X |  |  |
| *Self-care and participation* | Life space mobility | Alabama Life-Space Assessment (LSA)^15^ | X^2^ |  | X^2^ | X^2^ |  |
|  |  | Nursing home life space diameter^16^ | X^2^ |  | X^2^ | X^2^ |  |
|  | Self-care ability | Barthel Index^17^ | X |  |  |  |  |
| *Health Status and Economics* | Quality of life | EQ-5D-3L^18^ | X |  | X | X |  |
|  | Osteoporosis-related quality of life | Quality of Life Questionnaire of the European Foundation for Osteoporosis: QUALEFFO-41 (in T0 only subscale ADL, iADL)^19^ | X |  | X | X |  |
|  | Fracture related pain | Self-developed questionnaire | X |  | X | X |  |
|  | Pain intensity (during activity and inactivity) | Numeric rating scale (NRS)^20^ | X | X | X | X |  |
|  | Frequency of pain 6 months before index fracture | Self-developed rating scale | X^2^ |  |  |  |  |
|  | Body composition | Bioelectrical Impedance Analysis - BIA (AKERN BIA 101 N/H, SMT medical GmbH & Co. KG, Würzburg, D) | X |  |  |  |  |
|  | Use of professional or non-professional health service | Self-developed questionnaire | X^2^ |  | X^2^ | X^2^ |  |
|  | Current place of being (at home / short-term care/nursing home/hospital/rehabilitation | Self-developed questionnaire |  |  | X^2^ | X^2^ |  |
| *Physical capacity/ performance* | Physical performance | de Morton Mobility Index (DEMMI)^21^ | X |  | X |  |  |
|  |  | Short Physical Performance Battery (SPPB)^22^ |  |  | X |  |  |
|  | Walking ability | New mobility score (NMS)^23,24^ | X^2^ |  | X | X |  |
|  |  | Self-developed questionnaire | X^2^ |  | X | X |  |
|  | Handgrip strength | Dynamometer (Jamar, Saehan Corporation, South Korea) | X |  | X |  |  |
| *Physical activity* | Sensor-based measurement of physical activity over 7 days | ActivPAL4 micro (PAL Technologies, Glasgow, UK) |  |  | X |  |  |
| *Biomaterial* | Blood and urine sample | EDTA Full Blood 2,7 ml | X^3^ | X^3^ |  |  |  |
|  |  | EDTA Plasma 7,5 ml | X^3^ | X^3^ |  |  |  |
|  |  | Serum 4,7 ml | X^3^ | X^3^ |  |  |  |
|  |  | Urine Sample 10-12 ml | X^3^ | X^3^ |  |  |  |

S1 Fig. SPIRIT schedule_FriDA
