# Supplementary material for: Older patients with vertebral and pelvic fractures: Study protocol of a clinical cohort
Source: PLoS One. 2024 Aug 27;19(8):e0306727. doi: 10.1371/journal.pone.0306727 (PMC11349230; doi:10.1371/journal.pone.0306727)
Supplement: S1 File — (PDF) [file pone.0306727.s002.pdf]

DeepL
Subscribe to DeepL Pro to edit this document.
Visit www.DeepL.com/pro for more information.

Study plan / examination plan

Post-inpatient development of the ability to perform daily activities in elderly patients after fracture of the spine and pelvis

Study code: S00611

~~11.12.2020~~

Version 3 from 23/02/2021

Amendment 1 from 23/02/2021

Participants and responsibility

Study director and medically responsible: Prof. Dr Clemens Becker, Chief Physician of the Department of Geriatrics and Clinic for Geriatric Rehabilitation at Robert-Bosch-Krankenhaus Stuttgart, Auerbachstr. 110, 70376 Stuttgart, Tel.: 0711/81013101, Email: clemens.becker@rbk.de
Prof. Dr Bernd Kinner, Chief Physician of the Department of Orthopaedics and Trauma Surgery, Robert-Bosch-Krankenhaus Stuttgart, Auerbachstr. 110, 70376 Stuttgart, Tel.: 0711/8101-6012, Email: bernd.kinner@rbk.de
Project coordination: Prof. Dr Kilian Rapp, Senior Physician and Deputy Head of Research in the Department of Geriatrics and Clinic for Geriatric Rehabilitation at the Robert Bosch Hospital Stuttgart, Auerbachstr. 110, 70376 Stuttgart, Tel.: 0711/8101-5846, Email: kilian.rapp@rbk.de
Contact person for the Ethics Committee: Dr Ulrich Lindemann, Clinic for Geriatric Rehabilitation at the Robert-Bosch-Krankenhaus Stuttgart, Auerbachstr. 110, 70376 Stuttgart, Tel.: 0711/81012231, Fax: 0711/81013199, Email: ulrich.lindemann@rbk.de
Project staff: Oliver Schmitt, Senior Physician, Department of Orthopaedics and Trauma Surgery, Robert Bosch Hospital, Auerbachstraße 110, 70376 Stuttgart, phone 0711/8101-6012, email: oliver.schmitt@rbk.de
Miklos Lovasz, Assistant Physician, Department of Orthopaedics and Trauma Surgery, Robert Bosch Hospital, Auerbachstrasse 110, 70376 Stuttgart, phone 0711/8101-2033, email: miklos.lovasz@rbk.de
Michaela Groß, Physiotherapist, Clinic for Geriatric Rehabilitation at Robert-Bosch-Krankenhaus Stuttgart, Auerbachstr. 110, 70376 Stuttgart, Tel.: 0711/8101-3175, Email: michaela.gross@rbk.de
Rebekka Leonhardt, Physiotherapist, Clinic for Geriatric Rehabilitation at the Robert Bosch Hospital Stuttgart, Auerbachstr. 110, 70376 Stuttgart, Tel.: 0711/8101-6074, Email: rebekka.leonhardt@rbk.de
Statistical advice: Prof. Dr Jochen Klenk, Clinic for Geriatric Rehabilitation at the Robert Bosch Hospital Stuttgart, Auerbachstr. 110, 70376 Stuttgart, Germany
Tel.: 0711/81015853, Fax: 0711/81013199, Email: jochen.klenk@rbk.de and
Institute of Epidemiology and Medical Biometry, University of Ulm, Helmholtzstr. 22, 89081 Ulm

Biobank analyses: Prof Dr Matthias Schwab, Dr Margarete Fischer-Bosch Institute for Clinical Pharmacology, Auerbachstraße 112, 70376 Stuttgart, phone 0711/81013700, email: matthias.schwab@ikp-stuttgart.de

Financing

The study is being financed from the Robert Bosch Hospital's own funds.

Table of contents

Table of contents 3
1. list of abbreviations 3
2. basics 4
3. summarising the study 6
4. duration of study 7
5 Study population 7
6 Study procedure and examination methods 8
7. biobank 12
8 Planned methods 13
9 Evaluation strategy 17
10 Risks and side effects 19
11 Cancellation criteria 19
12. data protection 20
13 Ethical issues 21
14 Informing the study participants 22
15. plants 22
16. signatures 23
17 Literature 24

1. list of abbreviations

ATZ Ageing Trauma Centre
NAZ- Emergency Centre
R-taken from the routine database of the RBK
RBK-Robert-Bosch-Krankenhaus GmbH
RBMF Robert Bosch Gesellschaft für medizinische Forschung mbH

2. basics

2.1 Introduction/Problems

The hip fracture is the prototype of an osteoporotic fragility fracture. It is usually treated as an inpatient and is therefore easy to record using routine data. Therefore, a large number of observational studies on the epidemiology of hip fractures are available (Rapp et al., 2019). In addition to studies that utilise routine data, several specific hip fracture cohorts have also been established that allow more detailed conclusions to be drawn about the consequences of hip fractures (Autier et al., 2000; Cumming et al., 1996; Magaziner et al., 2003; Orwig et al., 2018). Therefore, the evidence regarding incidence (Icks et al., 2013) and consequences of hip fractures (Dyer et al., 2016) is high.

The data situation is significantly worse for other fragility fractures. One reason for this is that they are more difficult to collect via routine data (incomplete recording). In addition, surgical treatment requiring hospitalisation has only increased significantly for several fracture types in recent years. Of particular interest here are vertebral body and pelvic fractures.

Spinal and pelvic fractures are characterised by a considerable burden of disease and are typical fragility fractures in geriatric traumatology. It is not always known whether a fall alone is responsible or whether other factors are responsible for or favour the occurrence of a fracture. Furthermore, it is not known whether certain fracture types / fracture localisations are associated with certain causes of falls. A structured, evidence-based or guideline-supported assessment of falls is rarely carried out. Conservative and surgical procedures compete for treatment. There is little data on medium and long-term outcomes, which are generally not substantiated by controlled studies. Mobility, in particular the assessment of physical performance and physical activity, has hardly been investigated to date. Therefore, the description of fall histories in the context of possible correlations with certain fracture types and fracture localisations is desirable in future cohort studies. Furthermore, relevant outcomes for cohorts following spinal and pelvic fractures should be evaluated in preparation for intervention studies.

2.2 State of knowledge

Almost one in two women and one in four to five men over the age of 50 can expect to suffer a fragility fracture during the rest of their lives (Kanis et al., 2000).

The annual incidence of vertebral fractures in Europe is estimated at 520,000 cases (Hernlund et al., 2013). Older age and female gender are considered to be risk factors (Ong et al., 2018). No reliable data on the incidence or prevalence of vertebral fractures is available for Germany. Falls are a common cause of spinal injuries in older people (Cummings and Melton (Cummings and Melton, 2002). The relationship between certain causes of falls and the localisation of the fracture (cervical, thoracic, lumbar spine) has not yet been described. However, vertebral

fractures are not always associated with falls, but can also be the result of manifest osteoporosis (Ong et al., 2018). In this case, vertebral fractures often remain undetected. Those affected suffer from back pain, have limited mobility or are increasingly dependent on support in everyday life without realising that a vertebral fracture is the cause of their health condition (Ong et al., 2018; Ross, 1997). Fragility fractures, such as vertebral fractures, are also associated with nutritional deficiencies or sarcopenia. Sarcopenia is a risk factor for fragility fractures, particularly in males, if there is also low bone density. (Wong et al., 2019). Outcomes reported to date include mortality, the post-hospital setting, pain and the need for care/independence (Ong et al., 2018).

A review describes an incidence rate for pelvic fractures of 22.4/10,000 person-years for over 60-year-olds in Germany (Andrich et al., 2015). Different fracture types can be distinguished in terms of localisation (anterior/posterior pelvic girdle, unilateral/bilateral) and complexity (e.g. non-displaced/displaced) (Oberkircher et al., 2018). Women are also more frequently affected by pelvic fractures than men and the risk increases with age. Falls are the most common cause of pelvic fractures. The correlation between certain causes of falls and certain fracture types has not yet been described. Age, general state of health and fracture type are cited as criteria for deciding between conservative and surgical treatment (Höch et al., 2019). Mobility and pain have been described as relevant outcomes (Oberkircher et al., 2018).

Although mobility-associated outcomes are considered relevant for both case groups, studies to date have refrained from providing a detailed description of mobility, both in terms of physical performance and physical activity and participation.

2.3 Own preparatory work and possibilities

Similar to the planned procedure, corresponding studies on relevant outcomes have already been conducted and successfully published in the case group of hip fractures (Benzinger et al., 2019; Rapp et al., 2019; Schulz et al., 2019). The expertise to date is essentially based on the epidemiology of fractures and the implementation of intervention studies. To this end, 2 BMBF-funded consortium projects on osteoporotic fractures have been led:

- PROFinD 1: Prevention and rehabilitation of osteoporotic fractures in disadvantaged populations (2010 - 2014; funding code: 01EC1007A)
- PROFinD 2: Prevention and rehabilitation of osteoporotic fractures in disadvantaged populations (2015 - 2019; funding code: 01EC1404A)

As a result, there are templates for expanding the spectrum to include spinal and pelvic fractures.

Around 200 vertebral fractures and 120 pelvic fractures are seen each year in the Emergency Centre (NAZ) of the Trauma Surgery Department at the Robert Bosch Hospital (RBK) and receive further treatment in the Geriatric Trauma Centre (ATZ).

Since mid-2019, the routine examinations for these case groups in the NAC and the ATZ have been extensively expanded in order to prepare for the establishment of cohorts in these case groups. Patients (aged over 70) with a fracture of the spine or pelvis are contacted after admission to the ATZ. The interview includes a precise determination of the origin of the fall. The acute medical procedure and various patient characteristics are already recorded via the routine data.

3. summarising the study

In patients with spinal and pelvic fractures, outcomes relating to quality of life, participation, need for assistance, functionality/mobility, pain and fear of falling are to be assessed in two medium-term follow-up examinations (4 and 12 months after admission to the NAC). To derive predictive parameters, routine data is used that was previously collected in the RBK's NACC and in the subsequent care departments. When collecting the routine data, the precise description of a possible fall as the cause of the current fracture is of prominent importance for describing possible correlations with certain fracture types and localisations.

3.1 Aims of the study

The aim of the study is to establish a clinical cohort including a biodatabase that records patients with incisional vertebral body and pelvic fractures. The following questions are in the foreground:

- What percentage of (clinically treated and hospitalised) vertebral fractures can be attributed to falls? (retrospective analysis)
- Which localisations of the spine are particularly caused by falls (or osteoporosis)? (retrospective analysis)
- Which biomechanical fall mechanisms contribute to a vertebral or pelvic fracture? (retrospective analysis)
- Which parameters contribute positively and negatively to functionality, mobility, falls and/or impairment or participation in patients with vertebral or pelvic fractures? (prospective analysis)
- Development of an age trauma register for the fracture entities vertebral body and pelvic fracture
- Establishment of a biodatabase to complement the clinical study and investigate the biological causes of abnormalities in the post-inpatient development of the ability to perform daily activities in elderly patients following spinal and pelvic fractures. For example, osteology is a relevant topic in geriatric medicine for the consequences of falls, but also for delayed rehabilitation. For example, bone-relevant biomarkers are to be determined and the bone status and turnover of the affected patients analysed.

Other aspects, such as nutrition and/or medication intake, can have a significant influence on the post-inpatient development of the ability to perform everyday activities in older patients. Analyses of the metabolome and drug concentrations can help to better understand such causes. The necessary methods are described in detail on page 14.

The collected biomaterials, together with the clinical data, are available exclusively for scientific questions in compliance with data protection regulations.

The collected biomaterials are only used for projects that are submitted again to the responsible ethics committee for independent advice.

3.2 Clinical relevance of the planned study

Spinal and pelvic fractures are frequently treated fractures in German hospitals. Compared to hip fractures, the epidemiology of these two fragility fractures has been little studied and can benefit from this study. The identification of relevant outcomes can improve the assessment of the post-inpatient course (quality assurance). Furthermore, interventions can be developed which can then be evaluated in controlled studies. In the long term, this will serve to improve care in both case groups.

4. duration of study

Patients have already been included in the study since 2020 (vote of the Ethics Committee of the University of Tübingen dated 16 January 20202; reference 879/2019BO2). With this amendment, it should be possible to collect additional biomaterials and set up a biodatabase.

This is an open cohort. An a priori fixed time limit is not envisaged.

In addition, further study centres are to be recruited to participate in the development of the cohort. An ethics vote will be obtained from the relevant ethics committees of the cooperating study centres.

5. study population

For this cohort study, men and women who are admitted to the RBK's ATZ due to a (sub)acute spinal or pelvic fracture within the last 3 months are approached (i.e. inclusion criteria). A further inclusion criterion is the willingness to undergo a follow-up examination after 4 and 12 months (see below). Exclusion criteria are a) age ≤ 70 years, b) terminal illnesses, c) pathological fractures, d) severe dysarthria, aphasia, e) severe mental/psychiatric illness, f) insufficient knowledge of German, g) insufficient hearing ability, g) impaired cognition (according to initial examination, see below), h) place of residence outside the Stuttgart city area, i) no telephone accessibility and j) no independent ability to walk before study-related fracture event.

No one is obliged to participate in this study. The patient information makes it clear that refusal to participate in the study or withdrawal from the study at any time will have no adverse consequences for the patient's further medical or medical care. All patients are also informed of their right to withdraw their consent at any time without giving reasons.

6 Study procedure and examination methods

For the planned cohort study, 2 follow-up examinations are to be conducted at T2 and T3. The T2 follow-up examination is carried out in the participant's own home (flat or care home). The total duration of the examination protocol, which takes place 4 months after admission to the NAC, is around 60 minutes in total and can be split into two appointments if necessary. The T3 follow-up examination, which is carried out 12 months after admission to the NAC, takes place by telephone.

The data collected in this way is supplemented by routine data from the initial examinations at the RBK (this is regulated in the standard patient contract).

Initial examination in the ATZ of the RBK:

1. The initial examination (T0) is carried out as early as possible during the inpatient stay in the patient's room. As part of this initial examination (T0) in the ATZ, data is also taken from the patient file so that the interview takes a maximum of 30 minutes.
2. The discharge examination (T1) is carried out shortly before discharge from the ATZ and contains only a few parameters that can be taken from the patient file, as well as a maximum 5-minute interview.

The parameters described below are collected during the follow-up examinations or taken from the RBK routine database (R). Table 1 provides an overview of the examinations and measurement times.

Description of the collective

- Age (R), height (R), weight (R, T3) are recorded to describe the collective.
- In order to recognise changes in the level of care, this is assessed several times (R and T2, T3).
- The level of education is described by the number of years of schooling and training. (R)
- The result of the cognition test used in the RBK is taken from the patient file to assess the corresponding exclusion criterion. In weighted scores, temporal orientation, working memory and short-term memory are assessed with up to 28 error points

(Short Orientation Memory Concentration Test (Katzman et al., 1983)). It is dichotomised into cognitively impaired and non-cognitively impaired. (R)

- The living situation before the event is recorded via the type of housing (e.g. care home, living independently, etc.) and whether the participant lives alone or not. (R) . These parameters are also recorded at T2/ T3. In addition, the planned place of residence (R) and the actual place of residence (if different from the living situation) are recorded (R, T2, T3).
- The health status of the participants is measured by the Charlson Comorbidity Index (Charlson et al., 1987) recorded. In a standardised survey, 16 illnesses are documented with a weighted score. The total value is calculated for the description. In addition, other injuries/diseases requiring treatment are documented. (R) The subjectively perceived general state of health is also recorded (R, T, T3)
- The ten-year risk of a future fracture is determined using the Fracture Risk Assessment Tool (Middleton et al., 2012) calculated . Clinical risk factors (a previous clinically conspicuous fracture in adulthood, a hip fracture in a parent, nicotine consumption, use of cortisone, rheumatoid arthritis, other systemic disease with the risk of secondary osteoporosis, such as insulin-dependent diabetes mellitus), alcohol consumption and the result of a bone densitometry are taken into account. (R)
- The performance of a bone density measurement and (if applicable) the result is recorded retrospectively (R, T1, T2).
- When documenting the fall as a possible cause of the fracture, the event is described in terms of location, time and cause. In addition, the sequence of movements is described and the patient is asked about previous falls in the previous 12 months. (R)
- Falls are documented using a falls calendar in the period from discharge from ATZ to T2 and T2 to T3. Patients receive the monthly sheets at T1. Falls are also categorised in terms of fall location (indoor/outdoor), activity before the fall and consequences of the fall (serious, moderate, none).
- A bioelectrical impedance analysis (AKERN BIA 101 N/H, SMT medical GmbH & Co. KG, Würzburg, Germany) is carried out to determine body composition. A distinction is made between fat mass [%] and lean mass (body cell mass, extracellular mass) [%] with regard to the body compartments. (R)

Medical care and aftercare

- To describe the medical treatment, a distinction is made between surgical and conservative treatment. In addition, the localisation of the fracture, the fracture type and additional injuries requiring treatment (in relation to the time of the study-relevant event) are documented. (R, T2, T3)

- The number of days of inpatient acute care is recorded. (R)
- The number of days of possible inpatient rehabilitation and/or short-term care are recorded in the period from discharge from the outpatient treatment centre to T2 or T2 to T3. (T2, T3)
- The number of outpatient therapy units (physiotherapy, occupational therapy, medical training therapy, active group therapy) in the period from discharge from ATZ to T2 and T2 to T3 is recorded. (T2, T3)
- The extent and type of help [days/week] in everyday life (household and/or care, formal/informal) during the last week before the event (R) and in the period from discharge to T2 and T2 to T3 (T2, T3) is recorded.
- Whether an osteoporosis medication was prescribed and actually taken before admission to the NAC is recorded retrospectively (R). The type and dosage of the prescription and actual intake of pain and osteoporosis medication is recorded at the follow-up examinations T2 and T3. (T2, T3)

Quality of life and neuro-psychiatric assessment

- Quality of life is measured using the EuroQol 5-dimensional questionnaire (Rabin and de Charro, 2001) and the Quality of Life Questionnaire-41 (van Schoor et al., 2006) is recorded. The EuroQol 5-dimensional questionnaire covers the areas of mobility, independence, general activities, pain/discomfort and anxiety/depression with a 3-point scale and also includes a visual analogue scale (0-100) to describe the state of health. The Quality of Life Questionnaire contains 41 questions with a 3-5 scale on the areas of pain, activities of daily living, activities in the home, exercise, leisure and social activities, perception of general health and mood. The raw values of the total score and the domain scores are transformed to a 0-100 scale. Either only the subscales on (informal) activities of daily living (R) or the total are used (T2, T3).
- Fall-associated self-efficacy is assessed with a 1-item question (R) and with the Falls Efficacy Scale International (short version) (Yardley et al., 2005) recorded. It asks about 16 everyday activities in relation to concerns about falling. The 4-point scaled response options range from "no concerns at all" (1) to "very serious concerns" (4). (T 2, T3)
- Depressiveness is measured using the Depression in Old Age Scale (Heidenblut and Zank, 2010) recorded. Depressiveness is expressed using a total score of 10 questions (yes/no). (R, T2, T3)
- Pain is assessed using a numerical rating scale from 0 (no pain) to 10 (very severe pain). A distinction is made between pain at rest and pain on exertion. (R, T2, T3) In addition, the frequency of pain before the event is asked about (no pain, rarely, frequently, daily). (R)

Independence and mobility

- Independence in activities of daily living is measured using the Barthel Index (Mahoney and Barthel, 1965) recorded. Ten abilities (eating/drinking, bathing/showering, personal hygiene, dressing/dressing, stool control, urine control, using the toilet, bed/chair transfer, independent walking, climbing stairs) are rated with 0-15 points, with a possible total score of 100 points. (R)
- Mobility before the fracture event is assessed retrospectively or current mobility at the time of the follow-up interview using the New Mobility Score (Parker and Palmer, 1993) is surveyed. Mobility in the home and outside and shopping are rated on a 4-point scale from 0 = "not possible at all", 1 = "possible with personal assistance", 2 = "possible with aids" to 3 = "without problems". (R, T2, T3) The subjectively perceived change is also assessed (T2, T3).
- The participants' radius of action is measured using the University of Alabama Life-Space Assessment (Baker et al., 2003) recorded. The spatial radius of activity, the frequency of activity and the use of aids are each categorised. A total score of 120 points can be achieved. The radius of activity of participants living in a nursing home is measured using the Nursing Home Life-Space Diameter (Tinetti and Ginter, 1990) recorded. The spatial radius of activity and the frequency of activity are categorised. A total score of 50 points can be achieved. (R, T2, T3)
- Current mobility and physical performance are measured using the De Morton Mobility Index (de Morton et al., 2008) and the Short Physical Performance Battery (Guralnik et al., 1994) are used. In the De Morton Mobility Index, 15 abilities in the areas of bed (3 questions), chair (3 questions), static balance (4 questions), walking (2 questions) and dynamic balance (3 questions) are assessed with 0 = "not possible", 1 = "possible" or 2 = "independent". The raw score (x/19) is transformed into a score (x/100). In addition, the type of aid used (indoor/outdoor) is asked. (R, T2)
- Physical performance is measured using the Short Physical Performance Battery (SPPB) (Guralnik et al., 1994) is used. The SPPB tests balance in a standing position (closed stance, semi-tandem stance, tandem stance), standing up and sitting down 5 times from/on a chair and walking speed over 2.44 metres using a stopwatch. Individual scores (0-4) and a total score (0-12) are calculated for the 3 tests on the basis of reference values. (T2)
- Hand force is measured using a dynamometer (Jamar, Saehan Corporation, South Korea). The participant sits on a chair for the measurement. The elbow is bent at 90°. The dynamometer is held in the hand. The person is asked to squeeze the device as

tightly as possible. Three tests are carried out with the left and right hand. The highest value of the right and left hand is recorded. (R, T2)

- To describe physical activity, an activity monitor (activPAL4 micro, PAL Technologies, Glasgow, UK; Figure 1) is attached to the front of one thigh during the follow-up visits (T2) at the end of the first visit. The sensor, which is attached with a skin-friendly/medical film, is waterproof and should be worn for 7 whole days. The activity monitor is removed after 8 days. The activity monitor measures the daily cumulative walking, activity and inactivity time. The time of getting up in the morning and going to bed in the evening is noted for each day in a documentation of the daily rhythm. (T2)

Figure 1: activPAL 4 micro activity monitor

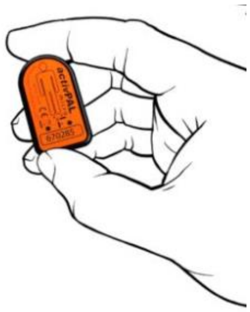

- The participant's conviction and motivation with regard to physical self-exercise is recorded using three individual questions. (T2/ T3)
- The extent of subjectively perceived social support is measured using the Oslo Social Support Scale (Delgard, 1996) measured. The scale contains three questions. It asks about the number of people the person can rely on, the extent of sympathy from other people and how easy it is for the person to get practical help from neighbours. The individual scores from the three questions are added together to form a score, which can range from 3 to 14 points. (T2, T3)

7. biobank

It is planned to collect additional biomaterial from patients as part of the clinical visits. After informing and obtaining the patient's consent to collect additional biomaterial, biomaterial should generally be collected at the planned times of T0 and T1. In exceptional cases, biomaterials may also be collected at the follow-up visit (T2). The following biomaterials are primarily considered: blood, urine, saliva, possibly stool.

Blood samples

For study-related reasons, a single blood sample of 2 x 9 ml (EDTA whole blood) should be taken from all participants. If possible, the study-related blood sample should be taken as part of a routine blood collection in order to avoid an additional venipuncture. Residual blood samples from patients that can no longer be used can also be utilised. The blood sample can be used for genetic, biochemical, metabolic and protein analyses. For this purpose, the sample is further processed accordingly (see below).

Saliva

Saliva is a very good alternative to blood if, for example, genetic tests are planned or certain biochemical markers, including drug concentrations, are to be measured. It is also possible to collect saliva from older patients; chewing sugar-free chewing gum may be useful for a short time before collecting saliva.

Urine

A spontaneous urine sample should be collected.

Chair

Prepared sample material is available for stool samples, which can also be used to collect a stool sample after discharge if necessary. If the stool sample is collected at home, patients are given a pre-paid envelope on discharge with which the sample can be sent.

Labelling and storage of samples

The collected biomaterials are labelled with a pseudonymised sample code and stored for an indefinite period of time. The reason for the long storage period is that numerous targets (e.g. in the genome, transcriptome, proteome, metabolome, etc.) are to be examined with the available sample material, but the analyses for this are in most cases very complex and can therefore take several years. For this reason, it must also be ensured that existing clinical data linked to the biomaterials can be utilised. The unlimited storage of the material and its use is explicitly pointed out to patients when they are informed and their consent is requested.

8 Planned methods

The biomaterials required for the following analyses (e.g. blood, urine, saliva, stool) should be collected and preserved according to standardised protocols.

The analyses should generally be carried out at the IKP Stuttgart. In the event that the corresponding methodology is not available at the IKP, the samples will also be sent to national and international cooperation partners for further analyses in pseudonymised form only. This also applies to methods that have not yet been developed and will be established in the course

of this project. Complex data can be analysed in the same way with external partners. Patients are informed in detail about the various necessary data protection regulations and the resulting consequences in the patient information leaflet.

Methods to be applied using the biomaterial:

Genetic analyses using DNA/RNA

Genomic DNA is isolated from the sample material (e.g. blood, saliva) using standard methods (e.g. standardised DNA isolation kits) and stored at 4°C in the IKP until molecular biological analysis. In the same way, RNA is isolated from the sample material using standardised methods, which also enable the determination of specific non-coding RNAs (e.g. miRNAs). Genetic variants (both common and rare) are detected using established methods such as real-time PCR methods, mass spectrometric detection (MALDI-TOF MS technology) or nanofluid technology. In the case of gene analyses at RNA level, standardised methods (e.g. real-time RT-PCR methods) are also used. In addition, it is also possible to use DNA/RNA micro-arrays, which allow more comprehensive analyses of the human genome. Analyses of the complete genome (i.e. complete sequencing of DNA/RNA) are also possible. The IKP in Stuttgart has extensive knowledge of these methods. The data is analysed according to a standardised procedure using bioinformatic expertise.

Epigenetic analyses

Epigenetic analyses (e.g. DNA methylation, chromatin methylation, miRNA and His-tone acetylation) are carried out using standardised assays/methods. Individual analyses, micro-arrays and more complex technologies are also used here. The data is evaluated according to a standardised procedure using bioinformatic expertise.

Proteomics

Detection of proteins or peptide patterns in biomaterials should be carried out using standardised methods (e.g. mass spectrometry). The data will be analysed according to a standardised procedure using bioinformatics expertise.

Metabolomics

Endogenous metabolites or metabolite profiles as well as drug concentrations (taking into account degradation products, so-called metabolites) can be detected using various mass spectrometric methods as well as biochemical assays. In the case of metabolomics, the link to publicly available metabolite databases enables structural elucidation and thus identification

of metabolites that could not previously be correctly assigned to known substances. The data is analysed according to a standardised procedure using bioinformatics expertise.

Microbiome

The microbiome analysis of a stool sample enables the description of bacteria that colonise the intestine. The method for this is usually the sequencing of the metagenome using standardised procedures. In rare cases, the cultivation of stool samples can also be useful for microbiome analyses. The data is analysed according to a standardised procedure using bioinformatic expertise.

Table 1: Parameters and measuring instruments

**Parameters**	**Instrument(s) / Variables**	**T0**	**T1**	**T2**	**T3**
Personalised[R]	Date of birth, gender	X			
	Height/weight, BMI	X			X[1]
	Level of education (years of schooling/training)	X			
	Housing situation	X[2]		X	X
	Living alone yes/no	X		X	X
	Place of residence (if different from living situation)		X	X	X
	Degree of care	X		X	X
	General state of health	X		X	X
Routine data stay [R]	Admission/discharge date	X	X		
	Surgical/conservative treatment		X	X [2]	X [2]
	(Fracture) diagnoses (ICD + subclassifications)	X		X[2]	X[2]
	Additional injuries requiring treatment	X			
	Comorbidities (Charlson)	X			
Fracture risk	FRAX	X			
Bone density[R]	DXA, T-Score (if available)	X		X	X
Fall anamnesis, fall evaluation	History of falls (falls relevant to the study)	X			
	Accident mechanism (study-relevant fall)	X			
	Falls retrospective	X[2]			
	Falls prospectively (fall diary)		X	X	X
Care after discharge	Hospital and rehabilitation days			X[2]	X [2]
	KZP - Days			X [2]	X[2]
	Assistance in everyday life (related to 1 week, type and frequency, formal/informal)	X[2]		X[2]	X [2]
	Extent of perceived social support (OSSS-3)			X[2]	X [2]
Physical exercises/ therapy	Conviction and motivation regarding physical exercises Exercises (3 questions)			X	X
	Physiotherapy/occupational therapy, active exercise group/MTT training (units since discharge)			X [2]	X[2]
Medication	Pain medication, yes/no, categorisation according to WHO (prescribed and taken in the last 7 days)			X	X
	Osteoporosis medication[R]	X[2]		X	X
Action area	University of Alabama Life-Space Assessment or Nursing Home Life-Space Diameter	X[2]		X[2]	X[2]
Pain	Intensity - NRS (rest, exercise)	X		X	X
	Chronic pain	X[2]			
Depressiveness[R]	DIA-S	X		X	X

Cognition[R]	BOMCT	X			
Fall-associated self-efficacy	FES-I short			X	X
	1-item question	X			
Perceived change	Minimal important difference (MID)			X	X
Quality of life	EQ-5D	X		X	X
Quality of life	QUALEFFO-41 (subscales ADL, iADL)	X		X	X
Independence (ADL)[R]	Barthel	X			
Mobility[R]	DEMMI	X		X	
	New Mobility Score*, walking ability with/without aids (indoor/outdoor)	X[2]		X	X
	Additional question on walking ability / aids	X[2]		X	X
Functional performance	SPPB, incl. walking speed			X	
	Manual force	X		X	
Sarcopenia screening	Bioelectrical impedance analysis (BIA)	X			
Physical activity	ActivPal (7 days, e.g. walking time/day)			X	
	Daily rhythm (during the ActivPal system)			X	
Organic materials e.g. Blood, urine, saliva and faeces		X[3]	X[3]	X[3]	

[1] Weight only
[R] usually Routine data/ to be recorded via chart review
[2] Retrospective recording
[3] according to test plan, if possible at time T0 or T1 but possibly also later at T2
T0= 2nd-4th day after admission to geriatric traumatology
T1 = time shortly before discharge
T2 = 120 days after hospitalisation

BOMCT = Blessed Orientation-Memory-Concentration Test (German version) (acute group)
DEMMI = De Morton Mobility Index
DIA-S = The Depression in Old Age Scale
FES-I = Falls Efficacy Scale - International (German version)
FRAX = Fracture Risk Assessment Tool (German version)
NRS = Numerical Rating Scale (pain at rest and during exercise)
EQ-5D = EuroQol-5 Dimensions Questionnaire
QUALEFFO-41 = Quality of Life Questionnaire of the European Foundation for Osteoporosis (German version)
SPPB = Short Physical Performance Battery

9. evaluation strategy

1. What percentage of (clinically treated and hospitalised) vertebral fractures can be attributed to falls?

To answer this question, a detailed questionnaire was created to clarify in the interview with the patient whether the complaints were preceded by a fall. This is not always clear-cut. Therefore, in addition to the questions, the study staff also assess the probability of a fall or no fall being the cause of the symptoms. The survey is retrospective. The analyses are purely descriptive in nature (frequencies).

2. Which localisations on the spine are particularly caused by falls (or osteoporosis)?

This question is based on the same data as 1). Due to the moderate number of cases, this question serves to generate hypotheses. The analyses are also descriptive in nature (frequencies), stratified for the fracture localisations on the spine.

3. Which biomechanical fall mechanisms contribute to a vertebral body or pelvic fracture?

To this end, the interview attempts to reconstruct the fall mechanism (initial fall direction; direction and localisation of the primary impact) as accurately as possible. The analyses are descriptive in nature (frequencies). A comparison with biomechanical data on fall mechanisms in hip fracture is planned. On the one hand, video data from Canada (not yet published) is available for this purpose. On the other hand, this information will be collected in an EU-funded study starting in 2021. The Robert Bosch Hospital is one of the study centres.

4. Which parameters contribute positively and negatively to functionality, mobility, impairment and participation in patients with vertebral body or pelvic fractures?

This is a classic prospective approach of a cohort study in which predictors for relevant endpoints such as functionality (e.g. walking speed), mobility (e.g. cumulative sensor-based walking time), impairment (e.g. limitation in activities of daily living) and participation (e.g. social outdoor activity) are analysed. Multivariate methods (e.g. linear or logistic regression) are used for the analyses.

5. Development of an age trauma register for the fracture entities vertebral body and pelvic fracture

The study approach is used to test the validity and feasibility of variables of an age trauma register for the fracture entities vertebral body and pelvic fractures. The register is based on the existing DGU® Age Trauma Register for hip fractures, but is modified to include specific aspects of vertebral body and pelvic fractures.

6. The establishment of a database with biomaterials is also planned. The phenotypically well-characterised cohort will be used to answer further questions in the field of geriatric traumatology and geriatric medicine. The preservation of biomaterials is planned for this purpose.

10 Risks and side effects

All the measurement instruments used to collect the data described above have already been used in other studies in comparable geriatric populations (e.g. after hip fracture). This showed that no risks and/or side effects are to be expected for the patients.
The devices used in the study have been tested for safety (systems). They are used in accordance with their authorised purpose and are only operated, used and maintained by persons who have the necessary training, knowledge and experience.

Possible risks and complications of blood sampling

With the additional collection of 2 x 9 ml from the venous system, no significant haemodynamic stress is to be expected, even in elderly patients.
In very rare cases, thromboses, incorrect punctures, the accidental puncture of an artery or nerve or infections are possible as a result of blood sampling. Depending on the nerve affected, nerve damage can have a wide range of possible consequences, from temporary pain, short-term paralysis and numbness to chronic, uncontrollable pain or permanent paralysis.
In rare cases, local vein irritation, superficial vein inflammation or the formation of a local haematoma may occur, which usually disappears after a few days without further therapeutic measures.

With regard to a benefit/risk assessment, there is no direct benefit for the participants from taking part in the data collection. However, conspicuous examination results will be passed on to the general practitioner after consultation and with the consent of the participants. In view of the potential social benefit with the future possibility of predicting adverse events and the possibility of intervention (e.g. if there is a risk of falling) and a very low risk for the participants, we believe that the data collection is justified.

11. cancellation criteria

Data collection may be cancelled if complications occur or at the express request of the participants. Such complications may take any form and degree of discomfort or anxiety if this occurs as a result of the examination or for any other reason at the time of the examination. Participants may also withdraw their consent at any time without giving reasons. In the event of withdrawal from the study, participants can decide for themselves whether existing data may continue to be used or must be deleted.
If unforeseen events occur that contribute to the disadvantage of the participants due to the study, the study management (i.e. medically responsible person) decides to discontinue the study.

The study management also reserves the right to terminate the study prematurely for individual participants if the state of health of the test person requires this. However, measured values collected up to this point can be used for analysis.

12. data protection

All project staff are subject to a duty of confidentiality. The information sheet and declaration of consent are adapted to the applicable General Data Protection Regulation. The patient data collected in the study will be treated confidentially by those responsible and protected against unauthorised access. Prof Rapp is named as the person responsible for data collection in this study at the RBK and for data processing in the context of the biodatabase. The project manager of the study centre is named as the person responsible for data collection at the respective study centre.

Patients are informed about this in a separate patient information leaflet and are asked to provide biomaterials for additional scientific investigations within the framework of a biodatabase. Furthermore, the study participants are informed that the biomaterials become the property of Robert-Bosch-Krankenhaus GmbH and Robert Bosch Gesellschaft für medizinische Forschung mbH when they are transferred to the biodatabase.

Consent for the biodatabase is voluntary and does not affect participation in the above-mentioned study.

The documentation of the collected data and its archiving is pseudonymised in a protected study file to which only authorised employees, including doctoral students bound to professional and data secrecy, have access.

All data is pseudonymised and stored at the Robert Bosch Hospital. For this purpose, an ID number is assigned to each person's data record. A list linking ID numbers and names is only available to the study management and the project coordination of the trial centre for possible withdrawals and data deletions. The written records are kept in a locked room and destroyed after 10 years. The data will be made publicly available in anonymous form after the end of the study for scientific analysis if required. Personal data, such as names and contact information, will remain at the study centre and will not be passed on. All participants will be informed about how the data will be handled and analysed.

If the patient withdraws consent, the patient can decide voluntarily whether data already collected up to that point can continue to be stored and used. If the patient explicitly requests the deletion of the data, the data must be deleted completely.

In the event that the patient expressly wishes the sample material to be destroyed even after consent has been given, the sample is assigned to the patient concerned and destroyed on the basis of the identification list kept.

All personal and clinical data collected and all biomaterials obtained will be stored for an indefinite period. The data will be anonymised no later than 10 years after consent to participate in the study has been given. It is not possible to withdraw consent or to destroy data and samples after anonymisation, and study participants will be informed of this.

The data collected as part of the study can also be used and further processed for future research projects at the clinic or institute.

The transfer of data to third parties (universities, research institutes and research companies for the purposes of medical research) as part of the study evaluation and other analyses of the results is pseudonymised and only to the extent necessary. Under certain circumstances, this also includes the transfer of data for research projects abroad or to co-operation partners outside the European Economic Area, i.e. to countries with a lower level of data protection (e.g. the USA). In such cases, it is agreed, as far as legally possible, that the co-operation partners are obliged to comply with the EU level of data protection. Nevertheless, it cannot be ruled out in every case that all conditions will be met exactly and that independent supervisory authorities may not be available to support the participant in exercising their data subject rights. Patients will be informed of this.
When data is passed on to external researchers, it is pseudonymised twice.
The research results from the study are published in anonymised form in specialist journals or in scientific databases.
Study participants are informed in the patient information that they can request information about their stored data at any time (including the provision of a free copy) and have the right to have incorrect data corrected. Study participants can also request that their data be deleted or anonymised at any time and can contact the data protection officer of the Robert Bosch Hospital and/or the Federal Data Protection Commissioner or the State Data Protection Commissioner in the event of complaints.
The legal basis for the processing of your data is Art. 6, 7, 9, 89 of the General Data Protection Regulation in conjunction with §§ 22, 27, 29, 32, 33, 34, 35, 36, 38 in the version of the EU Data Protection Adaptation and Implementation Act of 30 June 2017, Federal Law Gazette I, p. 2097 ff.
Note for test centres subject to the LDSG:
The legal basis for the processing of your data is Art. 6, 7, 9, 89 of the General Data Protection Regulation in conjunction with §§ 4, 5, 6, 8, 9, 12, 13 of the Baden-Württemberg State Data Protection Act in the version applicable from 25 May 2018.

13 Ethical issues

The study will be conducted in accordance with good clinical practice and in compliance with the principles of the 1964 Declaration of Helsinki and all subsequent revisions. All participants

must sign a written declaration of consent. Professional counselling of the study by the responsible ethics committee is carried out. No persons will be included in the study until the ethics committee has presented its favourable assessment of the procedures. The project management will take the recommendations of the ethics committee into account.

14. informing the study participants

All potential participants are approached at the RBK. After expressing interest, they are informed verbally and in writing about the study and the possibility of participating. A copy of the study information and a declaration of consent and data protection declaration are handed over and remain with the interested party. All participants are informed that participation or non-participation has no influence on treatment at the RBK. They will also be explicitly informed that RBK routine data will be included in the study. Before the first follow-up examination, the consent and data protection declaration must be signed by the participants. Before signing and before the first measurement, additional information will be provided in which any final questions from the participants will be answered.

Parts of the study, such as the use of biomaterials, can also be rejected without this resulting in exclusion from the remaining parts of the study.

15. plants

- Information about the study for interested parties
- Declaration of consent
- Patient information and declaration of consent for the use of biomaterials and associated data in the biodatabase
- Data collection forms
- Description/safety certifications of the methods used

16. signatures

Stuttgart, 23 February 2021 Prof Dr Clemens Becker
Chief Physician at the Clinic for Geriatric Rehabilitation,
Robert Bosch Hospital, Stuttgart

Stuttgart, 23.02.2021 Prof. Dr med. Bernd Kinner
Chief Physician of the Department of Orthopaedics and Trauma Surgery,
Robert Bosch Hospital Stuttgart

17. literature

Andrich, S., Haastert, B., Neuhaus, E., Neidert, K., Arend, W., Ohmann, C., Grebe, J., Vogt, A., Jungbluth, P., Rösler, G., Windolf, J., Icks, A., 2015. Epidemiology of Pelvic Fractures in Germany: Considerably High Incidence Rates among Older People. PloS One 10, e0139078. https://doi.org/10.1371/journal.pone.0139078

Autier, P., Haentjens, P., Bentin, J., Baillon, J.M., Grivegnée, A.R., Closon, M.C., Boonen, S., 2000. Costs induced by hip fractures: a prospective controlled study in Belgium. Belgian Hip Fracture Study Group. Osteoporos. Int. J. Establ. Result Coop. Eur. Found. Osteoporos. Natl. Osteoporos. Found. USA 11, 373-380. https://doi.org/10.1007/s001980070102

Baker, P.S., Bodner, E.V., Allman, R.M., 2003. Measuring life-space mobility in community-dwelling older adults. J. Am. Geriatr. Soc. 51, 1610-1614. https://doi.org/10.1046/j.1532-5415.2003.51512.x

Benzinger, P., Riem, S., Bauer, J., Jaensch, A., Becker, C., Büchele, G., Rapp, K., 2019. Risk of institutionalisation following fragility fractures in older people. Osteoporos. Int. J. Establ. Result Coop. Eur. Found. Osteoporos. Natl. Osteoporos. Found. USA 30, 1363-1370. https://doi.org/10.1007/s00198-019-04922-x

Charlson, M.E., Pompei, P., Ales, K.L., MacKenzie, C.R., 1987. A new method of classifying prognostic comorbidity in longitudinal studies: development and validation. J. Chronic Dis. 40, 373-383. https://doi.org/10.1016/0021-9681(87)90171-8

Cumming, R.G., Klineberg, R., Katelaris, A., 1996. Cohort study of risk of institutionalisation after hip fracture. Aust. N. Z. J. Public Health 20, 579-582. https://doi.org/10.1111/j.1467-842x.1996.tb01069.x

Cummings, S.R., Melton, L.J., 2002. epidemiology and outcomes of osteoporotic fractures. Lancet Lond. Engl. 359, 1761-1767. https://doi.org/10.1016/S0140-6736(02)08657-9

de Morton, N.A., Davidson, M., Keating, J.L., 2008. The de Morton Mobility Index (DEMMI): an essential health index for an ageing world. Health Qual. Life Outcomes 6, 63. https://doi.org/10.1186/1477-7525-6-63

Delgard, O.S., 1996, Community health profile: A tool for psychiatric prevention, in: Trend, D.R., Reed, C.A. (Eds.), Promotion of Mental Health. Aldershot, Avebury, pp. 395-402.

Dyer, S.M., Crotty, M., Fairhall, N., Magaziner, J., Beaupre, L.A., Cameron, I.D., Sherrington, C., Fragility Fracture Network (FFN) Rehabilitation Research Special Interest Group, 2016. A critical review of the long-term disability outcomes following hip fracture. BMC Geriatr. 16, 158. https://doi.org/10.1186/s12877-016-0332-0

Guralnik, J.M., Simonsick, E.M., Ferrucci, L., Glynn, R.J., Berkman, L.F., Blazer, D.G., Scherr, P.A., Wallace, R.B., 1994. A short physical performance battery assessing lower extremity function: association with self-reported disability and prediction of mortality and nursing home admission. J. Gerontol. 49, M85-94. https://doi.org/10.1093/geronj/49.2.m85

Heidenblut, S., Zank, S., 2010 [Development of a new screening instrument for geriatric depression. The depression in old age scale (DIA-S)]. Z. Gerontol. Geriatr. 43, 170-176. https://doi.org/10.1007/s00391-009-0067-z

Hernlund, E., Svedbom, A., Ivergård, M., Compston, J., Cooper, C., Stenmark, J., McCloskey, E.V., Jönsson, B., Kanis, J.A., 2013. Osteoporosis in the European Union: medical management, epidemiology and economic burden. A report prepared in collaboration with the International Osteoporosis Foundation (IOF) and the European Federation of Pharmaceutical Industry Associations (EFPIA). Arch. Osteoporos. 8, 136. https://doi.org/10.1007/s11657-013-0136-1

Höch, A., Pieroh, P., Gras, F., Hohmann, T., Märdian, S., Holmenschlager, F., Keil, H., Palm, H.-G., Herath, S.C., Josten, C., Schmal, H., Stuby, F.M., Pelvic Injury Register of the German Trauma Society, 2019. age and "general health"-beside fracture classification-affect the therapeutic decision for geriatric pelvic ring fractures: a

German pelvic injury register study. Int. orthop. https://doi.org/10.1007/s00264-019-04326-w
Icks, A., Arend, W., Becker, C., Rapp, K., Jungbluth, P., Haastert, B., 2013. incidence of hip fractures in Germany, 1995-2010. arch. Osteoporos. 8, 140. https://doi.org/10.1007/s11657-013-0140-5
Kanis, J.A., Johnell, O., Oden, A., Jonsson, B., De Laet, C., Dawson, A., 2000. Risk of hip fracture according to the World Health Organisation criteria for osteopenia and osteoporosis. Bone 27, 585-590.
Katzman, R., Brown, T., Fuld, P., Peck, A., Schechter, R., Schimmel, H., 1983. Validation of a short Orientation-Memory-Concentration Test of cognitive impairment. Am. J. Psychiatry 140, 734-739. https://doi.org/10.1176/ajp.140.6.734
Magaziner, J., Fredman, L., Hawkes, W., Hebel, J.R., Zimmerman, S., Orwig, D.L., Wehren, L., 2003. Changes in functional status attributable to hip fracture: a comparison of hip fracture patients to community-dwelling aged. Am. J. Epidemiol. 157, 1023-1031. https://doi.org/10.1093/aje/kwg081
Mahoney, F.I., Barthel, D.W., 1965. FUNCTIONAL EVALUATION: THE BARTHEL INDEX. Md State Med J. 14, 61-65.
Middleton, R.G., Shabani, F., Uzoigwe, C.E., Shoaib, A., Moqsith, M., Venkatesan, M., 2012. FRAX and the assessment of the risk of developing a fragility fracture. J. Bone Joint Surg. Br. 94, 1313-1320. https://doi.org/10.1302/0301-620X.94B10.28889
Oberkircher, L., Ruchholtz, S., Rommens, P.M., Hofmann, A., Bücking, B., Krüger, A., 2018. Osteoporotic Pelvic Fractures. German. Arzteblatt Int. 115, 70-80. https://doi.org/10.3238/arztebl.2018.0070
Ong, T., Kantachuvesiri, P., Sahota, O., Gladman, J.R.F., 2018. Characteristics and outcomes of hospitalised patients with vertebral fragility fractures: a systematic review. Ageing 47, 17-25. https://doi.org/10.1093/ageing/afx079
Orwig, D., Hochberg, M.C., Gruber-Baldini, A.L., Resnick, B., Miller, R.R., Hicks, G.E., Cappola, A.R., Shardell, M., Sterling, R., Hebel, J.R., Johnson, R., Magaziner, J., 2018. Examining Differences in Recovery Outcomes between Male and Female Hip Fracture Patients: Design and Baseline Results of a Prospective Cohort Study from the Baltimore Hip Studies. J. Frailty Aging 7, 162-169. https://doi.org/10.14283/jfa.2018.15
Parker, M.J., Palmer, C.R., 1993. A new mobility score for predicting mortality after hip fracture. J. Bone Joint Surg. Br. 75, 797-798.
Rabin, R., de Charro, F., 2001. EQ-5D: a measure of health status from the EuroQol Group. Ann. Med. 33, 337-343. https://doi.org/10.3109/07853890109002087
Rapp, K., Büchele, G., Dreinhöfer, K., Bücking, B., Becker, C., Benzinger, P., 2019. Epidemiology of hip fractures : Systematic literature review of German data and an overview of the international literature. Z. Gerontol. Geriatr. 52, 10-16. https://doi.org/10.1007/s00391-018-1382-z
Ross, P.D., 1997 Clinical consequences of vertebral fractures. Am. J. Med. 103, 30S-42S; discussion 42S-43S. https://doi.org/10.1016/s0002-9343(97)90025-5
Schulz, C., Büchele, G., Rehm, M., Rothenbacher, D., Roigk, P., Rapp, K., Günster, C., König, H.-H., Reber, K., 2019. Patient Characteristics as Indicator for Care Dependence after Hip Fracture: A Retrospective Cohort Study Using Health Insurance Claims Data From Germany. J. Am. Med. Dir. Assoc. 20, 451-455.e3. https://doi.org/10.1016/j.jamda.2018.09.029
Tinetti, M.E., Ginter, S.F., 1990. The nursing home life-space diameter. A measure of extent and frequency of mobility among nursing home residents. J. Am. Geriatr. Soc. 38, 1311-1315. https://doi.org/10.1111/j.1532-5415.1990.tb03453.x
van Schoor, N.M., Knol, D.L., Glas, C. a. W., Ostelo, R.W.J.G., Leplège, A., Cooper, C., Johnell, O., Lips, P., 2006. Development of the Qualeffo-31, an osteoporosis-specific quality-of-life questionnaire. Osteoporos. Int. J. Establ. Result Coop. Eur. Found. Osteoporos. Natl. Osteoporos. Found. USA 17, 543-551. https://doi.org/10.1007/s00198-005-0024-7

Wong, R.M.Y., Wong, H., Zhang, N., Chow, S.K.H., Chau, W.W., Wang, J., Chim, Y.N., Leung, K.S., Cheung, W.H., 2019. The relationship between sarcopenia and fragility fracture-a systematic review. Osteoporos. Int. J. Establ. Result Coop. Eur. Found. Osteoporos. Natl. Osteoporos. Found. USA 30, 541-553. https://doi.org/10.1007/s00198-018-04828-0

Yardley, L., Beyer, N., Hauer, K., Kempen, G., Piot-Ziegler, C., Todd, C., 2005. Development and initial validation of the Falls Efficacy Scale-International (FES-I). Ageing 34, 614-619. https://doi.org/10.1093/ageing/afi196
