# Supplementary material for: Older patients with vertebral and pelvic fractures: Study protocol of a clinical cohort
Source: PLoS One. 2024 Aug 27;19(8):e0306727. doi: 10.1371/journal.pone.0306727 (PMC11349230; doi:10.1371/journal.pone.0306727)
Supplement: S2 File — (PDF) [file pone.0306727.s003.pdf]

Studienplan / Prüfplan

Poststationäre Entwicklung der Alltagsfähigkeit bei älteren Patienten nach Fraktur an der Wirbelsäule und am Becken

Studiencode: S00611

~~11.12.2020~~

Version 3 vom 23.02.2021

Amendment 1 vom 23.02.2021

Teilnehmer und Verantwortlichkeit

Studienleitung und medizinisch verantwortlich: Prof. Dr. med. Clemens Becker, Chefarzt der Abteilung Geriatrie und Klinik für Geriatrische Rehabilitation im Robert-Bosch-Krankenhaus Stuttgart, Auerbachstr. 110, 70376 Stuttgart, Tel.: 0711/81013101, Email: clemens.becker@rbk.de

Prof. Dr. med. Bernd Kinner, Chefarzt der Abteilung Orthopädie und Unfallchirurgie, im Robert-Bosch-Krankenhaus Stuttgart, Auerbachstr. 110, 70376 Stuttgart, Tel.: 0711/8101-6012, Email: bernd.kinner@rbk.de

Projektkoordination: Prof. Dr. Kilian Rapp, Oberarzt und stellvertretender Leiter der Forschung in der Abteilung Geriatrie und Klinik für Geriatrische Rehabilitation im Robert-Bosch-Krankenhaus Stuttgart, Auerbachstr. 110, 70376 Stuttgart, Tel.: 0711/8101-5846, Email: kilian.rapp@rbk.de

Ansprechpartner für die Ethikkommission: Dr. Ulrich Lindemann, Klinik für Geriatrische Rehabilitation im Robert-Bosch-Krankenhaus Stuttgart, Auerbachstr. 110, 70376 Stuttgart, Tel.: 0711/81012231, Fax: 0711/81013199, Email: ulrich.lindemann@rbk.de

Projektmitarbeiterinnen und Projektmitarbeiter: Oliver Schmitt, Funktionsoberarzt, Abteilung für Orthopädie und Unfallchirurgie, Robert-Bosch-Krankenhaus, Auerbachstraße 110, 70376 Stuttgart, Telefon 0711/8101-6012, Email: oliver.schmitt@rbk.de

Miklos Lovasz, Assistenzarzt, Abteilung für Orthopädie und Unfallchirurgie, Robert-Bosch-Krankenhaus, Auerbachstraße 110, 70376 Stuttgart, Telefon 0711/8101-2033, Email: miklos.lovasz@rbk.de

Michaela Groß, Physiotherapeutin, Klinik für Geriatrische Rehabilitation im Robert-Bosch-Krankenhaus Stuttgart, Auerbachstr. 110, 70376 Stuttgart, Tel.: 0711/8101-3175, Email: michaela.gross@rbk.de

Rebekka Leonhardt, Physiotherapeutin, Klinik für Geriatrische Rehabilitation im Robert-Bosch-Krankenhaus Stuttgart, Auerbachstr. 110, 70376 Stuttgart, Tel.: 0711/8101-6074, Email: rebekka.leonhardt@rbk.de

Statistische Beratung: Prof. Dr. Jochen Klenk, Klinik für Geriatrische Rehabilitation im Robert-Bosch-Krankenhaus Stuttgart, Auerbachstr. 110, 70376 Stuttgart

Tel.: 0711/81015853, Fax: 0711/81013199, Email: jochen.klenk@rbk.de und

Institut für Epidemiologie und medizinische Biometrie, Universität Ulm, Helmholtzstr. 22, 89081 Ulm

Biobankuntersuchungen: Prof. Dr. Matthias Schwab, Dr. Margarete Fischer-Bosch-Institut für Klinische Pharmakologie, Auerbachstraße 112, 70376 Stuttgart, Telefon 0711/81013700, Email: matthias.schwab@ikp-stuttgart.de

Finanzierung

Die Studie wird aus Eigenmitteln des Robert-Bosch-Krankenhauses finanziert.

Inhaltsverzeichnis

Inhaltsverzeichnis ... 3
1. Abkürzungsverzeichnis ... 3
2. Grundlagen ... 4
3. Zusammenfassende Beschreibung der Studie ... 6
4. Studiendauer ... 7
5. Studienpopulation ... 8
6. Studienablauf und Untersuchungsmethoden ... 8
7. Biobank ... 13
8. Geplante Methoden ... 15
9. Auswertungsstrategie ... 18
10. Risiken und Nebenwirkungen ... 20
11. Abbruchkriterien ... 21
12. Datenschutz ... 21
13. Ethische Belange ... 23
14. Aufklärung der Studienteilnehmer ... 23
15. Anlagen ... 24
16. Unterschriften ... 25
17. Literatur ... 26

1. Abkürzungsverzeichnis

ATZ - Alters-Trauma-Zentrum
NAZ - Notaufnahme-Zentrum
R - aus der Routinedatenbank des RBK entnommen
RBK - Robert-Bosch-Krankenhaus GmbH
RBMF Robert Bosch Gesellschaft für medizinische Forschung mbH

2. Grundlagen

2.1 Einleitung/Problematik

Die Hüftfraktur ist der Prototyp einer osteoporotischen Fragilitätsfraktur. Sie wird in der Regel stationär behandelt und ist somit einfach über Routinedaten zu erfassen. Deshalb liegt eine große Anzahl an Beobachtungsstudien zur Epidemiologie von Hüftfrakturen vor (Rapp et al., 2019). Neben Studien, die Routinedaten nutzen, wurden aber auch mehrere spezifische Hüftfrakturkohorten etabliert, die detailliertere Aussagen zu den Folgen von Hüftfrakturen erlaubten (Autier et al., 2000; Cumming et al., 1996; Magaziner et al., 2003; Orwig et al., 2018). Deshalb ist die Evidenz bezüglich Inzidenz (Icks et al., 2013) und Folgen von Hüftfrakturen (Dyer et al., 2016) hoch.

Deutlich schlechter ist die Datenlage bei anderen Fragilitätsfrakturen. Dies liegt unter anderem daran, dass sie schwieriger über Routinedaten zu erheben sind (unvollständige Erfassung). Außerdem hat die operative Versorgung, die einen stationären Aufenthalt erfordert, bei mehreren Frakturentitäten erst in den letzten Jahren deutlich zugenommen. Besonders interessant sind hierbei Wirbelkörper- und Beckenfrakturen.

Wirbelsäulen- und Beckenfrakturen sind gekennzeichnet durch eine erhebliche Krankheitslast und sind typische Fragilitätsfrakturen in der Alterstraumatologie. Ursächlich ist nicht immer bekannt, ob allein ein Sturz verantwortlich ist oder ob andere Faktoren für das Auftreten einer Fraktur verantwortlich sind bzw. diese begünstigen. Darüber hinaus ist nicht bekannt, ob bestimmte Frakturtypen / Frakturlokalisationen mit bestimmten Sturzhergängen assoziiert sind. Eine strukturierte, evidenzbasierte oder leitliniengestützte Abklärung des Sturzes erfolgt nur selten. Als Therapie konkurrieren konservative und operative Verfahren. Zu mittel- und langfristigen Outcomes gibt es wenige Daten, die i.d.R. nicht durch kontrollierte Studien belegt sind. Die Mobilität, insbesondere die Betrachtung der körperlichen Leistungsfähigkeit und die körperliche Aktivität, wurden dabei bisher kaum untersucht. Daher ist die Beschreibung der Sturzhergänge im Kontext möglicher Zusammenhänge mit bestimmten Frakturtypen und Frakturlokalisationen in zukünftigen Kohortenstudien wünschenswert. Weiterhin sollten relevante Outcomes für Kohorten nach Wirbelsäulen- und Beckenfrakturen zur Vorbereitung von Interventionsstudien evaluiert werden.

2.2 Stand des Wissens

Fast jede zweite Frau und jeder vierte bis fünfte Mann im Alter von 50 Jahren muss damit rechnen, im Laufe des restlichen Lebens eine Fragilitätsfraktur zu erleiden (Kanis et al., 2000).

Die jährliche Inzidenz von Wirbelkörperfrakturen wird für Europa mit 520.000 Fällen geschätzt (Hernlund et al., 2013). Dabei gelten ein höheres Alter und das weibliche Geschlecht als Risikofaktoren (Ong et al., 2018). Für Deutschland liegen keine belastbaren Daten zur Inzidenz

oder Prävalenz von Wirbelkörperfrakturen vor. Beim älteren Menschen sind Stürze eine häufige Ursachen für Verletzungen der Wirbelsäule (Cummings and Melton, 2002). Der Zusammenhang zwischen bestimmten Sturzhergängen und der Lokalisation der Fraktur (Hals-, Brust, Lendenwirbelsäule) wurde bisher noch nicht beschrieben. Wirbelkörperfrakturen sind jedoch nicht immer sturzassoziiert, sondern können auch das Ergebnis einer manifesten Osteoporose sein (Ong et al., 2018). In diesem Fall bleiben Wirbelkörperfrakturen oftmals unentdeckt. Betroffene leiden unter Rückenschmerzen, haben Einschränkungen in der Mobilität oder sind zunehmend auf Unterstützung im alltäglichen Leben angewiesen, ohne zu wissen, dass eine Wirbelkörperfraktur ursächlich für Ihren Gesundheitszustand ist (Ong et al., 2018; Ross, 1997). Fragilitätsfrakturen, wie Wirbelkörperfrakturen sie darstellen, gehen auch mit Ernährungsdefiziten oder einer Sarkopenie einher. Insbesondere für das männliche Geschlecht ist eine Sarkopenie ein Risikofaktor für eine Fragilitätsfraktur, wenn zusätzlich eine niedrige Knochendichte besteht. (Wong et al., 2019). Als Outcome wurde bisher über die Sterblichkeit, das post-stationäre Setting, Schmerz und die Pflegebedürftigkeit/Selbständigkeit berichtet (Ong et al., 2018).

In einer Übersichtsarbeit wird für über 60-jährige in Deutschland eine Inzidenzrate für Beckenfrakturen von 22,4/10.000 Personenjahre beschrieben (Andrich et al., 2015). Dabei können verschiedene Frakturtypen bezüglich Lokalisation (vorderer/hinterer Beckengürtel, unilateral/bilateral) und Komplexität (z.B. nicht-disloziert/disloziert) unterschieden werden (Oberkircher et al., 2018). Auch bei Beckenfrakturen sind Frauen häufiger betroffen als Männer und das Risiko steigt mit zunehmendem Alter an. Stürze sind die häufigste Ursache für Beckenfrakturen. Der Zusammenhang zwischen bestimmten Sturzhergängen und bestimmten Frakturtypen wurde bisher nicht beschrieben. Als Entscheidungskriterien zwischen konservativer und operativer Versorgung werden das Alter, der allgemeine Gesundheitszustand und der Frakturtyp genannt (Höch et al., 2019). Als relevante Outcomes wurden die Mobilität und Schmerzen beschrieben (Oberkircher et al., 2018).

Obwohl für beide Fallgruppen mobilitäts-assoziierte Outcomes als relevant erachtet werden, wurde bisher in Studien auf eine detaillierte Beschreibung der Mobilität, sowohl der körperlichen Leistungsfähigkeit, als auch der körperlichen Aktivität und Teilhabe, verzichtet.

2.3 Eigene Vorarbeiten und Möglichkeiten

Analog zum geplanten Vorgehen wurden in der Fallgruppe der Hüftfrakturen entsprechende Untersuchungen zu relevanten Outcomes bereits durchgeführt und erfolgreich publiziert (Benzinger et al., 2019; Rapp et al., 2019; Schulz et al., 2019). Die bisherige Expertise gründet im Wesentlichen auf der Epidemiologie von Frakturen und der Durchführung von Interventionsstudien. Dazu wurden 2 BMBF-geförderte Konsortialprojekte zu osteoporotischen Frakturen geleitet:

- PROFinD 1: Prävention und Rehabilitation osteoporotischer Frakturen in benachteiligten Populationen (2010 – 2014; Förderkennzeichen: 01EC1007A)
- PROFinD 2: Prävention und Rehabilitation osteoporotischer Frakturen in benachteiligten Populationen (2015 – 2019; Förderkennzeichen: 01EC1404A)

Dadurch bestehen Vorlagen zur Erweiterung des Spektrums auf Wirbelsäulen- und Beckenfrakturen.

Im Notaufnahme-Zentrum (NAZ) der Unfallchirurgie des Robert-Bosch-Krankenhauses (RBK) werden jährlich etwa 200 vertebrale Frakturen und 120 Beckenfrakturen gesehen und im Alters-Trauma-Zentrum (ATZ) weiter versorgt.

Seit Mitte 2019 wurden für diese Fallgruppen die Routineuntersuchungen im NAZ und im ATZ umfassend erweitert, um den Aufbau von Kohorten in diesen Fallgruppen vorzubereiten. Dabei werden Patient*innen (≥ 70 Jahre) mit Fraktur der Wirbelsäule oder des Beckens nach Aufnahme in das ATZ kontaktiert. Die Befragung beinhaltet ein genaues Eruieren des Sturzherganges. Das akutmedizinische Vorgehen und diverse Patientencharakteristika werden schon obligatorisch über die Routinedaten erfasst.

3. Zusammenfassende Beschreibung der Studie

Bei Patient*innen nach Wirbelsäulen- und Beckenfrakturen sollen in zwei mittelfristigen Nachuntersuchungen (4 und 12 Monate nach Aufnahme in das NAZ) Outcomes zu Lebensqualität, Teilhabe, Hilfsbedarf, Funktionalität/Mobilität, Schmerz und Sturzangst erhoben werden. Zur Ableitung prädiktiver Parameter werden Routinedaten genutzt, die zuvor im NAZ des RBK und in den anschließend versorgenden Abteilungen erhoben wurden. Bei der Erhebung der Routinedaten hat die genaue Beschreibung eines möglichen Sturzherganges als Ursache der aktuellen Fraktur eine prominente Bedeutung zur Beschreibung möglicher Zusammenhänge mit bestimmten Frakturtypen und -lokalisationen.

3.1. Ziele der Studie

Ziel der Studie ist der Aufbau einer klinischen Kohorte einschließlich einer Biodatenbank, die Patient*innen mit inzidenter Wirbelkörper- und Beckenfraktur erfasst. Folgende Fragestellungen stehen hierfür im Vordergrund:

- Wieviel Prozent der (klinisch apparenten und stationär behandelten) vertebralen Frakturen können auf Stürze zurückgeführt werden? (retrospektive Analyse)
- Welche Lokalisationen an der Wirbelsäule sind insbesondere durch Stürze (bzw. osteoporotisch) bedingt? (retrospektive Analyse)
- Welche biomechanischen Sturzmechanismen tragen zu einer Wirbelkörper- bzw. Beckenfraktur bei? (retrospektive Analyse)

- Welche Parameter tragen positiv und negativ zur Funktionalität, Mobilität, Stürzen und/oder Beeinträchtigung bzw. Teilhabe bei Patient*innen mit Wirbelkörper- bzw. Beckenfraktur bei? (prospektive Analyse)
- Entwicklung eines Alters-Trauma-Registers für die Frakturentitäten Wirbelkörper- und Beckenfraktur
- Aufbau einer Biodatenbank, mit der ergänzend zur Klinischen Studie biologische Ursachen für Auffälligkeiten bei der poststationären Entwicklung der Alltagsfähigkeit bei älteren Patient*innen nach Fraktur an der Wirbelsäule und am Becken untersucht werden sollen. Beispielhaft ist die Osteologie in der Altersmedizin ein relevantes Thema für Sturzkonsequenzen aber auch für eine verzögerte Rehabilitation. So sollen beispielsweise knochenrelevante Biomarker bestimmt und damit der Knochenstatus und -umsatz der betroffenen Patient*innen untersucht werden. Weitere Aspekte, wie z.B. die Ernährung und/oder auch Medikamenteneinnahme, können die poststationäre Entwicklung der Alltagsfähigkeit bei älteren Patient*innen erheblich beeinflussen. Untersuchungen des Metaboloms bzw. auch Arzneimittelkonzentrationen können dazu beitragen, solche Ursachen besser zu verstehen. Die notwendigen Methoden werden auf Seite 14 im Detail beschrieben.

Die gesammelten Biomaterialien stehen zusammen mit den klinischen Daten unter Einhaltung des Datenschutzes ausschließlich für wissenschaftliche Fragestellungen zur Verfügung.

Es werden mit den gesammelten Biomaterialien ausschließlich Projekte durchgeführt, die nochmals der zuständigen Ethik-Kommission zu einer unabhängigen Beratung vorgelegt werden.

3.2. Klinische Relevanz der geplanten Untersuchung

Wirbelsäulen- und Beckenfrakturen sind häufig behandelte Frakturen in deutschen Krankenhäusern. Im Vergleich zu Hüftfrakturen ist die Epidemiologie dieser beiden Fragilitätsfrakturen wenig untersucht und kann durch diese Studie profitieren. Die Identifizierung von relevanten Outcomes kann die Beurteilung des poststationären Verlaufs verbessern (Qualitätssicherung). Weiterhin können Interventionen entwickelt werden, die dann in kontrollierten Studien evaluiert werden können. Langfristig dient dies der Verbesserung in der Versorgung in den beiden Fallgruppen.

4. Studiendauer

Es werden bereits seit 2020 Patient*innen in die Studie eingeschlossen (Votum der Ethikkommission der Universität Tübingen vom 16.01.20202; Zeichen 879/2019BO2). Mit dem

vorliegenden Amendement soll es möglich sein, ergänzend Biomaterialien zu sammeln und eine Biodatenbank aufzubauen.
Es handelt sich um eine offene Kohorte. Eine a priori festgelegte zeitliche Limitierung ist nicht vorgesehen.
Es sollen zudem weitere Studienzentren dafür gewonnen werden, am Aufbau der Kohorte mitzuwirken. Ein Ethikvotum wird dafür bei den zuständigen Ethikkommissionen der jeweils kooperierenden Studienzentren eingeholt.

5. Studienpopulation

Für diese Kohortenstudie werden Männer und Frauen, die im ATZ des RBK wegen einer (sub)akuten Wirbelsäulen- oder Beckenfraktur innerhalb der letzten 3 Monate aufgenommen werden, angesprochen (i.e. Einschlusskriterien). Ein weiteres Einschlusskriterium ist die Bereitschaft zur Nachuntersuchung nach 4 und 12 Monaten (s.u.). Ausschlusskriterien sind a) Alter ≤ 70 Jahre, b) terminale Erkrankungen, c) pathologische Frakturen, d) schwere Dysarthrie, Aphasie, e) schwere psychische/psychiatrische Erkrankung, f) unzureichende Deutschkenntnisse, g) unzureichendes Hörvermögen, g) eingeschränkte Kognition (gemäß Eingangsuntersuchung, s.u.), h) Wohnort außerhalb des Stuttgarter Stadtgebiets, i) keine telefonische Erreichbarkeit und j) keine selbständige Gehfähigkeit vor studienbedingtem Frakturereignis.
Niemand wird dazu verpflichtet, an dieser Studie teilzunehmen. Die Patienteninformation stellt klar, dass eine Weigerung an der Studie teilzunehmen oder der Rücktritt von der Untersuchung zu einem beliebigen Zeitpunkt ohne nachteilige Folgen für die weitere medizinische oder ärztliche Versorgung des Patienten bleibt. Alle Patienten werden außerdem über ihr Recht, jederzeit ohne Angabe von Gründen die Einwilligung rückgängig zu machen, aufgeklärt.

6. Studienablauf und Untersuchungsmethoden

Für die geplante Kohortenstudie sollen 2 Follow-Up-Untersuchungen zu den Terminen T2 und T3 durchgeführt werden.
Die T2-Follow-Up-Untersuchung wird in der eigenen Häuslichkeit (Wohnung oder Pflegeheim) der Teilnehmer*innen durchgeführt. Die Gesamtdauer des Untersuchungsprotokolls, das 4 Monate nach Aufnahme in das NAZ erfolgt, beträgt jeweils insgesamt etwa 60 Minuten und kann bei Bedarf auf zwei Termine aufgeteilt werden.
Die T3-Follow-Up-Untersuchung, welche 12 Monate nach Aufnahme in das NAZ durchgeführt wird, findet telefonisch statt.
Die so erhobenen Daten werden durch Routinedaten der Eingangsuntersuchungen im RBK ergänzt (dies ist im Standard-Patientenvertrag geregelt).

Eingangsuntersuchung im ATZ des RBK:

1. Die Eingangsuntersuchung (T0) wird möglichst früh während des stationären Aufenthaltes im Patientenzimmer durchgeführt. Im Rahmen dieser Eingangsuntersuchung (T0) im ATZ werden auch Daten aus der Patientenakte übernommen, so dass die Befragung maximal 30 Minuten dauert.
2. Die Entlassuntersuchung (T1) wird kurz vor Entlassung aus dem ATZ durchgeführt und beinhaltet nur wenige Parameter, die aus der Patientenakte übernommen werden können, sowie eine max. 5-minütige Befragung.

Die im Folgenden beschriebenen Parameter werden bei den Follow-Up-Untersuchungen erhoben oder aus der Routinedatenbank des RBK entnommen (R). Tabelle 1 zeigt die Untersuchungen und Messzeitpunkte im Überblick.

Beschreibung des Kollektivs

- Zur Beschreibung des Kollektivs werden Alter (R), Größe (R), Gewicht (R, T3) erfasst.
- Um Veränderungen im Pflegegrad zuerkennen wird dieser mehrfach erhoben (R und T2, T3).
- Das Bildungsniveau wird über die Anzahl der Schul- und Ausbildungsjahre beschrieben. (R)
- Das Ergebnis des im RBK verwendeten Kognitionstests wird zur Beurteilung des entsprechenden Ausschlusskriteriums aus der Patientenakte übernommen. In gewichteten Scores wird die zeitliche Orientierung, das Arbeitsgedächtnis und das Kurzzeitgedächtnis mit bis zu 28 Fehlerpunkten (Short Orientation Memory Concentration Test (Katzman et al., 1983)) gescreent. Es wird in kognitiv eingeschränkt und nicht kognitiv eingeschränkt dichotomisiert. (R)
- Die Wohnsituation vor dem Ereignis wird über die Wohnform (z.B. Pflegeheim, selbständig lebend, o.a.) erfasst sowie darüber, ob die Teilnehmerin/ der Teilnehmer alleine lebt oder nicht. (R) . Diese Parameter werden zusätzlich bei T2/ T3 erhoben. Zusätzlich wird der geplante Aufenthaltsort (R) und der tatsächliche Aufenthaltsort (wenn abweichend von der Wohnsituation) erfasst (R, T2, T3).
- Der Gesundheitszustand der Teilnehmerinnen wird durch den Charlson Komorbiditäts-Index (Charlson et al., 1987) erfasst. In einer standardisierten Befragung werden 16 Erkrankungen mit einem gewichteten Score dokumentiert. Zur Beschreibung wird der Summenwert berechnet. Zusätzlich werden weitere behandlungswürdige Verletzungen/Erkrankungen dokumentiert. (R) Der subjektiv wahrgenommene allgemeine Gesundheitszustand wird ebenfalls erfasst (R, T, T3)

- Das Zehnjahresrisiko einer zukünftigen Fraktur wird über das Fracture Risk Assessment Tool (Middleton et al., 2012) berechnet . Dabei werden klinische Risikofaktoren (eine vorausgegangene klinisch auffällige Fraktur im Erwachsenenalter, eine Hüftfraktur bei einem Elternteil, Nikotinkonsum, Einnahme von Cortison, Rheumatoide Arthritis, andere systemische Erkrankung mit dem Risiko einer sekundären Osteoporose, wie z.B. insulinpflichtiger Diabetes mellitus), Alkoholkonsum und das Ergebnis einer Knochendichtemessung berücksichtigt. (R)
- Die Durchführung einer Knochendichtemessung und (falls zutreffend) das Ergebnis wird retrospektiv erfasst (R, T1, T2).
- Bei der Dokumentation des Sturzes als mögliche Ursache der Fraktur wird das Ereignis örtlich, zeitlich und ursächlich beschrieben. Zusätzlich wird der Bewegungsablauf beschrieben und es wird nach vorherigen Stürzen in den vorherigen 12 Monaten gefragt. (R)
- Im Zeitraum Entlassung aus ATZ bis T2 und T2 bis T3 werden Stürze per Sturzkalender dokumentiert. Patient*innen erhalten die Monatsblätter zum Zeitpunkt T1. Stürze werden zudem in Bezug auf Sturzort (indoor/outdoor), Aktivität vor dem Sturz sowie Sturzfolgen (schwerwiegend, mittelschwer, keine) klassifiziert.
- Eine Bioelektrische Impedanzanalyse (AKERN BIA 101 N/H, SMT medical GmbH & Co. KG, Würzburg, D) wird zur Bestimmung der Körperzusammensetzung durchgeführt. Dabei wird bezüglich der Körperkompartimente in Fettmasse [%] und Magermasse (Körperzellmasse, Extrazellulärmasse) [%] unterschieden. (R)

Medizinische Versorgung und Nachsorge

- Zur Beschreibung der medizinischen Versorgung wird in operative und konservative Versorgung unterschieden. Zusätzlich werden die Lokalisation der Fraktur, der Frakturtyp und zusätzliche behandlungsbedürftige Verletzungen (bezogen auf den Zeitpunkt des studienrelevanten Ereignisses) dokumentiert. (R, T2, T3)
- Es wird die Anzahl der Tage der stationären Akutversorgung erfasst. (R)
- Es werden die Anzahl der Tage einer möglichen stationären Rehabilitation und/oder Kurzzeitpflege im Zeitraum Entlassung aus ATZ bis T2, bzw. T2 bis T3 erfasst. (T2, T3)
- Es wird die Anzahl der ambulanten Therapieeinheiten (Physiotherapie, Ergotherapie, medizinische Trainingstherapie, aktive Gruppentherapie) im Zeitraum Entlassung aus ATZ bis T2 und T2 bis T3 erfasst. (T2, T3)
- Es wird der Umfang und die Art der Hilfe [Tage/Woche] im Alltag (Haushalt und/oder Pflege, formell/ informell) während der letzten Woche vor dem Ereignis (R) und im Zeitraum Entlassung bis T2, sowie T2 bis T3 erfasst (T2, T3).

- Ob ein Osteoporosemedikament vor der Aufnahme ins NAZ verschrieben und tatsächlich eingenommen wurde, wird retrospektiv erfasst (R). Es wird die Art und Dosierung der Verschreibung und tatsächlichen Einnahme von Schmerz- und Osteoporosemedikation bei den Nachuntersuchungen T2 und T3 erfasst. (T2, T3)

Lebensqualität und neuro-psychiatrisches Assessment

- Die Lebensqualität wird über den EuroQol 5-dimensional questionnaire (Rabin and de Charro, 2001) und dem Quality of Life Questionnaire-41 (van Schoor et al., 2006) erfasst. Der EuroQol 5-dimensional questionnaire erfasst die Bereiche Mobilität, Selbständigkeit, allgemeine Aktivitäten, Schmerz/Unannehmlichkeiten und Angst/Depression mit einer 3er Skalierung und beinhaltet zusätzlich eine visuelle Analog Skala (0-100) zur Beschreibung des Gesundheitszustandes. Der Quality of Life Questionnaire beinhaltet 41 Fragen mit 3-5er Skalierung zu den Bereichen Schmerzen, Aktivitäten des täglichen Lebens, Tätigkeiten im Haus, Bewegung, Freizeit und soziale Aktivitäten, Wahrnehmung der allgemeinen Gesundheit und Stimmung. Die Rohwerte von Gesamtscore und den Bereichscores werden auf eine 0-100 Skalierung transformiert. Es werden entweder nur die Subscalen zu (informellen) Aktvitäten des täglichen Lebens (R) oder der gesamte verwendet (T2, T3).
- Die sturzassoziierte Selbstwirksamkeit wird mit einer 1-item Frage (R) und mit der Falls Efficacy Scale International (short version) (Yardley et al., 2005) erfasst. Es wird nach 16 Alltagstätigkeiten in Bezug auf die Bedenken dabei hinzufallen gefragt. Die 4er-skalierten Antwortmöglichkeiten reichen von „keinerlei Bedenken" (1) bis „sehr große Bedenken" (4). (T 2, T3)
- Die Depressivität wird mit der Depression im Alter – Skala (Heidenblut and Zank, 2010) erfasst. Die Depressivität wird über einen Gesamtscore aus 10 Fragen (ja/nein) ausgedrückt. (R, T2, T3)
- Schmerzen werden mit einer numerischen Rating Skala von 0 (kein Schmerz) bis 10 (sehr starke Schmerzen) bewertet. Dabei wird nach Schmerzen in Ruhe und bei Belastung unterschieden. (R, T2, T3) Zusätzlich wird nach der Häufigkeit von Schmerzen vor dem Ereignis gefragt (keine Schmerzen, selten, häufig, täglich). (R)

Selbständigkeit und Mobilität

- Die Selbständigkeit bei Aktivitäten des täglichen Lebens werden mit dem Barthel-Index (Mahoney and Barthel, 1965) erfasst. Zehn Fähigkeiten (Essen/Trinken, Baden/Duschen, Körperpflege, An-/Ausziehen, Stuhlkontrolle, Harnkontrolle, Benutzung der Toilette, Bett-/Stuhltransfer, selbständiges Gehen, Treppen steigen)

werden mit 0-15 Punkten, bei einem möglichen Gesamtscore von 100 Punkten, bewertet. (R)

- Die Mobilität vor dem Frakturereignis wird retrospektiv bzw. die aktuelle Mobilität zum Zeitpunkt der Follow-Up-Befragung über den New Mobility Score (Parker and Palmer, 1993) erfragt. Dabei werden die Mobilität in der Wohnung und draußen und das Einkaufen mit einer 4er Skalierung von 0 = „gar nicht möglich", 1 = „möglich mit personeller Unterstützung, 2 = „möglich mit Hilfsmittel" bis 3 = „ohne Probleme" bewertet. (R, T2, T3) Die subjektiv wahrgenommene Veränderung wird ebenso erhoben (T2, T3).
- Der Aktionsradius der Teilnehmer*innen wird über das University of Alabama Life-Space Assessment (Baker et al., 2003) erfasst. Dabei werden der räumliche Aktivitätsradius, die Häufigkeit der Aktivität und der Hilfsmittelgebrauch jeweils kategorial erfragt. Ein Gesamtscore von 120 Punkten kann erreicht werden. Der Aktionsradius von in Pflegeheim lebenden Teilnehmerinnen und Teilnehmern wird über den Nursing Home Life-Space Diameter (Tinetti and Ginter, 1990) erfasst. Dabei werden der räumliche Aktivitätsradius sowie die Häufigkeit der Aktivität jeweils kategorial abgefragt. Ein Gesamtscore von 50 Punkten kann erreicht werden. (R, T2, T3)
- Die aktuelle Mobilität und körperliche Leistungsfähigkeit wird über den De Morton Mobilitäts Index (de Morton et al., 2008) und die Short Physical Performance Battery (Guralnik et al., 1994) erhoben. Beim De Morton Mobilitäts Index werden 15 Fähigkeiten in den Bereichen Bett (3 Fragen), Stuhl (3 Fragen), statisches Gleichgewicht (4 Fragen), Gehen (2 Fragen) und dynamisches Gleichgewicht (3 Fragen) mit 0 = „nicht möglich", 1 = „möglich" oder 2 = „selbständig" bewertet. Der Rohwert (x/19) wird in einen Score (x/100) transformiert. Zusätzlich wird die Art der benutzten Hilfsmittel (indoor/ outdoor) erfragt. (R, T2)
- Die körperliche Leistungsfähigkeit wird über die Short Physical Performance Battery (SPPB) (Guralnik et al., 1994) erhoben. Beim SPPB wird das Gleichgewicht im Stand (geschlossener Stand, Semi-Tandem-Stand, Tandem-Stand), das 5malige Aufstehen und Hinsetzen von/auf einem/n Stuhl und die Gehgeschwindigkeit über 2,44 Meter mit einer Stoppuhr getestet. Anhand von Referenzwerten werden für die 3 Tests Einzel-Scores (0-4) und ein Gesamtscore (0-12) berechnet. (T2)
- Die Handkraft wird mit einem Dynamometer (Jamar, Saehan Corporation, Süd Korea) gemessen. Zur Messung sitzt der/die Teilnehmer*in auf einem Stuhl. Der Ellbogen ist angelegt und 90° gebeugt. Der Dynamometer wird in der Hand gehalten. Die Person wird aufgefordert das Gerät möglichst fest zusammenzudrücken. Mit der linken und der

rechten Hand werden jeweils 3 Versuche durchgeführt. Der jeweils höchste Wert von rechter und linker Hand wird erfasst. (R, T2)

- Zur Beschreibung der körperlichen Aktivität wird bei den Nachuntersuchungen (T2) am Ende des jeweils ersten Besuchs ein Aktivitätsmonitor (activPAL4 micro, PAL Technologies, Glasgow, UK; Abbildung 1) an einem Oberschenkel frontal aufgeklebt. Der mit hautfreundlicher/medizinischer Folie aufgeklebte Sensor ist wasserdicht und soll 7 ganze Tage getragen werden. Der Aktivitätsmonitor wird nach 8 Tagen abgenommen. Durch den Aktivitätsmonitor wird die tägliche kumulative Geh-, Aktivitäts- und Inaktivitätszeit gemessen. In einer Dokumentation des Tagesrhythmus wird für jeden Tag die Zeit des morgendlichen Aufstehens und des abendlichen Zubettgehens notiert. (T2)

Abbildung 1: Aktivitätsmonitor activPAL 4 micro

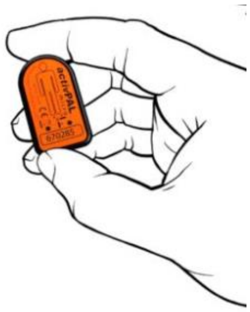

- Die Überzeugung sowie Motivation der Teilnehmerin bzw. des Teilnehmers hinsichtlich körperlicher Eigenübungen wird mittels dreier Einzelfragen erfasst. (T2/ T3)
- Das Ausmaß subjektiv wahrgenommener sozialer Unterstützung wird mit der Oslo Social Support Scale (Delgard, 1996) gemessen. Die Skala beinhaltet drei Fragen. Gefragt wird nach der Anzahl an Menschen, auf die sich die Person verlassen kann, das Ausmaß der Anteilnahme von anderen Menschen und wie einfach es für die Person ist, praktische Hilfe von Nachbarn zu erhalten. Durch Addition der Einzelpunktwerte aus den drei Fragen wird ein Score gebildet, der Werte zwischen 3 und 14 Punkten annehmen kann. (T2, T3)

7. Biobank

Im Rahmen der klinischen Visiten ist geplant, von den Patient*innen zusätzlich Biomaterial zu sammeln. Nach Aufklärung und Zustimmung der Patient*innenen Biomaterial zusätzlich sammeln zu dürfen, soll Biomaterial i.d.R zu den geplanten Zeitpunkten T0 bzw. T1 abgenommen werden. In Ausnahmefällen können auch bei der Follow-up Visite (T2)

Biomaterialien gesammelt werden. Als Biomaterialien kommen in erster Linie in Frage: Blut, Urin, Speichel, ggf. Stuhl.

Blutproben

Studienbedingt soll bei allen Teilnehmer*innen eine einmalige Blutentnahme von 2 x 9 ml (EDTA-Vollblut) durchgeführt werden. Die studienbedingte Blutentnahme sollte möglichst im Rahmen einer Routineblutabnahme erfolgen, um eine zusätzliche Venenpunktion zu vermeiden. Restblutproben der Patient*innen, die keine Verwendung mehr finden, können ebenfalls verwendet werden. Die Blutprobe kann für genetische, biochemische, metabolische und Proteinuntersuchungen verwendet werden. Dazu wird die Probe entsprechend weiter aufgearbeitet (siehe weiter unten).

Speichel

Speichel ist als Alternative zu Blut sehr gut zu verwenden, wenn z.B. genetische Untersuchungen geplant sind oder auch bestimmte biochemische Marker einschließlich Arzneimittelkonzentrationen gemessen werden sollen. Die Gewinnung von Speichel ist auch bei älteren Patient*innen gut möglich; ggf. kann vor der Speichelgewinnung kurzfristig das Kauen eines zuckerfreien Kaugummis sinnvoll sein.

Urin

Es soll eine Spontanurinprobe gesammelt werden.

Stuhl

Für Stuhlproben stehen vorbereitetes Probenmaterial zur Verfügung, womit eine Stuhlprobe ggf. auch nach Entlassung gesammelt werden soll. Wenn die Stuhlprobe zuhause gesammelt wird, bekommen die Patient*innen bei Entlassung einen vorfrankierten Rückumschlag mit, mit dem die Probe verschickt werden kann.

Kennzeichnung und Lagerung der Proben

Die gesammelten Biomaterialien werden mit einem pseudonymisierten Probencode gekennzeichnet und für einen unbefristeten Zeitraum aufbewahrt. Der Grund für die lange Aufbewahrungsdauer liegt darin, dass mit dem vorliegenden Probenmaterial zahlreiche Targets (z.B. im Genom, Transkriptom, Proteom, Metabolom, etc.) untersucht werden sollen, die Analysen dafür aber in den meisten Fällen sehr aufwendig sind und sich deshalb über mehrere Jahre hinziehen können. Aus diesem Grund muss auch sichergestellt werden, dass auf bereits vorliegende klinische, mit den Biomaterialien verknüpfte Daten zurückgegriffen

werden kann. Über die unbegrenzte Lagerung des Materials sowie deren Verwendung wird bei der Aufklärung der Patient*innen explizit hingewiesen und um ihre Zustimmung gebeten.

8. Geplante Methoden

Die für die nachstehenden Analysen notwendigen Biomaterialien (z.B. Blut, Urin, Speichel, Stuhl) sollen nach standardisierten Protokollen gesammelt und asserviert werden.
Die Analysen sollen i.d.R. im IKP Stuttgart durchgeführt werden. Für den Fall, dass die entsprechende Methodik im IKP nicht verfügbar ist, werden die Proben zu weiteren Untersuchungen auch an nationale und internationale Kooperationspartner ausschließlich pseudonymisiert versendet. Dies gilt auch für momentan noch nicht entwickelte Methoden, die im Verlaufe dieses Projektes neu etabliert werden. In gleicher Weise kann eine Auswertung komplexer Daten mit externen Partnern erfolgen. Über die unterschiedlichen notwendigen Datenschutzbestimmungen und sich daraus ergebenden Konsequenzen werden Patient*innen in der Patienteninformation ausführlich informiert.

Methoden, die unter Verwendung des Biomaterials angewendet werden sollen:

Genetische Untersuchungen unter Verwendung von DNA/RNA

Genomische DNA wird aus dem Probenmaterial (z.B. Blut, Speichel) mittels Standardmethoden (z.B. standardisierte DNA-Isolierungs-Kits) isoliert und bei 4°C bis zur molekularbiologischen Untersuchung im IKP aufbewahrt. In gleicher Weise wird RNA aus dem Probenmaterial mittels Standardmethoden isoliert, die auch eine Bestimmung von spezifischen nicht-kodierenden RNAs (z.B. miRNAs) ermöglicht.
Genetische Varianten (sowohl häufige wie seltene) werden mit etablierten Verfahren, wie z.B. Realtime-PCR Methoden, massenspektrometrische Detektion (MALDI-TOF MS Technologie) bzw. Nanofluid-Technologie detektiert. Im Falle von Genanalysen auf RNA-Ebene werden ebenfalls standardisierte Methoden (z.B. Realtime RT-PCR Methoden) verwendet. Zusätzlich ist es möglich, auch DNA/RNA Micro-Arrays zu verwenden, die umfangreichere Aussagen zum humanen Genom zulassen. Darüber hinaus sind Analysen des kompletten Genoms (d.h. Komplettsequenzierung der DNA/RNA) möglich. Umfassende Kenntnisse zu diesen Verfahren bestehen am IKP in Stuttgart. Die Auswertung der Daten erfolgt nach standardisiertem Vorgehen unter Verwendung bioinformatischer Expertise.

Epigenetische Analysen

Epigenetische Analysen (z.B. DNA-Methylierung, Chromatin-Methylierung, miRNA und Histon-Acetylierung) werden mittels standardisierter Assays/Methoden durchgeführt. Auch hier finden Einzelanalysen, Micro-Arrays bzw. komplexere Technologien Anwendung. Die

Auswertung der Daten erfolgt nach standardisiertem Vorgehen unter Verwendung bioinformatischer Expertise.

Proteomics

Detektion von Proteinen bzw. Peptidmustern in Biomaterialien soll mit standardisierten Methoden (z.B. Massenspektrometrie) durchgeführt werden. Die Auswertung der Daten erfolgt nach standardisiertem Vorgehen unter Verwendung bioinformatischer Expertise.

Metabolomics

Endogene Metabolite bzw. Metabolitenprofile sowie auch Arzneimittelkonzentrationen (unter Berücksichtigung von Abbauprodukten, sog. Metaboliten) können mit verschiedenen massenspektrometrischen Methoden aber auch biochemischen Assays detektiert werden. Im Falle von Metabolomics ist durch die Verknüpfung mit öffentlich verfügbaren Metabolitendatenbanken eine Strukturaufklärung und damit eine Identifizierung von Metaboliten möglich, die bisher nicht korrekt bereits bekannten Substanzen zugeordnet werden können. Die Auswertung der Daten erfolgt nach standardisiertem Vorgehen unter Verwendung bioinformatischer Expertise.

Microbiom

Die Mikrobiomanalyse einer Stuhlprobe ermöglicht die Beschreibung von Bakterien, die den Darm besiedeln. Die Methodik dafür ist i.d.R. die Sequenzierung des Metagenoms mittels standardisierter Verfahren. In seltenen Fällen kann für Mikrobiomuntersuchungen auch die Kultivierung der Stuhlproben sinnvoll sein. Die Auswertung der Daten erfolgt nach standardisiertem Vorgehen unter Verwendung bioinformatischer Expertise.

Tabelle 1: Parameter und Messinstrumente

Parameter	Instrument(e) / Variablen	T0	T1	T2	T3
Personenbezogen[R]	Geburtsdatum, Geschlecht	X			
	Größe/ Gewicht, BMI	X			X[1]
	Bildungsniveau (Schul-/ Ausbildungsjahre)	X			
	Wohnsituation	X[2]		X	X
	Alleinlebend ja/nein	X		X	X
	Aufenthaltsort (falls abweichend zu Wohnsituation)		X	X	X
	Pflegegrad	X		X	X
	Allgemeiner Gesundheitszustand	X		X	X
Routinedaten Aufenthalt[R]	Aufnahme-/Entlassdatum	X	X		
	Operative/ konservative Versorgung		X	X[2]	X[2]
	(Fraktur-) diagnosen (ICD + Subklassifikationen)	X		X[2]	X[2]
	Zusätzl. behandlungsbedürftige Verletzungen	X			
	Komorbiditäten (Charlson)	X			
Frakturrisiko	FRAX	X			
Knochendichte[R]	DXA, T-Score (sofern vorhanden)	X		X	X
Sturzanamnese, Sturzevaluation	Sturzanamnese (studienrelevanter Sturz)	X			
	Unfallmechanismus (studienrelevanter Sturz)	X			
	Stürze retrospektiv	X[2]			
	Stürze prospektiv (Sturztagebuch)		X	X	X
Versorgung nach Entlassung	Krankenhaus- und Rehabilitationstage			X[2]	X[2]
	KZP – Tage			X[2]	X[2]
	Hilfen im Alltag (bez. auf 1 Woche, Art und Häufigkeit, formell/informell)	X[2]		X[2]	X[2]
	Ausmaß an wahrgenommener sozialer Unterstützung (OSSS-3)			X[2]	X[2]
Körperliche Übungen/ Therapie	Überzeugung und Motivation bzgl. körperl. Übungen (3 Fragen)			X	X
	Physio-/Ergotherapie, aktive Übungsgruppe/MTT-Training (Einheiten seit Entlassung)			X[2]	X[2]
Medikamente	Schmerzmedikamente, ja/nein, Einstufung nach WHO (letzte 7 Tage verordnet sowie eingenommen)			X	X
	Osteoporosemedikation[R]	X[2]		X	X
Aktionsraum	University of Alabama Life-Space Assessment bzw. Nursing Home Life-Space Diameter	X[2]		X[2]	X[2]
Schmerzen	Intensität - NRS (Ruhe, Belastung)	X		X	X
	Chronische Schmerzen	X[2]			
Depressivität[R]	DIA-S	X		X	X

Kognition[R]	BOMCT	X			
Sturzassoziierte Selbstwirksamkeit	FES-I short			X	X
	1-item Frage	X			
Wahrgenommene Veränderung	Minimal important difference (MID)			X	X
Lebensqualität	EQ-5D	X		X	X
Lebensqualität	QUALEFFO-41 (Subskalen ADL, iADL)	X		X	X
Selbstständigkeit (ADL)[R]	Barthel	X			
Mobilität[R]	DEMMI	X		X	
	New Mobility Score*, Gehfähigkeit mit/ohne Hilfsmittel (indoor/outdoor)	X[2]		X	X
	Zusatzfrage Gehfähigkeit/ Hilfsmittel	X[2]		X	X
Funktionelle Leistungsfähigkeit	SPPB, inkl. Gehgeschwindigkeit			X	
	Handkraft	X		X	
Sarkopenie-Screening	Bioelektrische Impedanzanalyse (BIA)	X			
Körperliche Aktivität	ActivPal (7 Tage, z.B. Gehzeit/Tag)			X	
	Tagesrhythmus (während der ActivPal-Anlage)			X	
Bio-Materialien z.B. Blut, Urin, Speichel und Stuhl		X[3]	X[3]	X[3]	

[1] nur Gewicht
[R] in der Regel Routinedaten/ über Chart Review zu erfassen
[2] retrospektive Erfassung
[3] nach Prüfplan, wenn möglich zum Zeitpunkt T0 bzw T1 aber ggf. auch später zu T2
T0= 2.-4.Tag nach Aufnahme in Alterstraumatologie
T1 = Zeitpunkt kurz vor Entlassung
T2 = 120 Tage nach stationärer Aufnahme

BOMCT = Blessed Orientation-Memory-Concentration Test (deutsche Version) (Akutgruppe)
DEMMI = De Morton Mobility Index
DIA-S = Die Depression im Alter-Skala
FES-I = Falls Efficacy Scale – International (deutsche Version)
FRAX = Fracture Risk Assessment Tool (deutsche Version)
NRS = Numerische Ratingskala (Schmerz in Ruhe und bei Belastung)
EQ-5D = EuroQol-5 Dimensions Fragebogen
QUALEFFO-41 = Quality of Life Questionnaire of the European Foundation for Osteoporosis (deutsche Version)
SPPB = Short Physical Performance Battery

9. Auswertungsstrategie

1. Wieviel Prozent der (klinisch apparenten und stationär behandelten) vertebralen Frakturen können auf Stürze zurückgeführt werden?

Zur Beantwortung dieser Frage wurde ein detaillierter Fragebogen erstellt, der im Interview mit den Patient*innen klären soll, ob den Beschwerden ein Sturz vorausgegangen ist. Dies ist nicht immer eindeutig zu klären. Deshalb erfolgt zusätzlich zu den Fragen eine Bewertung durch das Studienpersonal, mit welcher Wahrscheinlichkeit ein bzw. kein Sturz Ursache der

Beschwerden ist. Die Befragung ist retrospektiv. Die Analysen sind rein deskriptiver Natur (Häufigkeiten).

2. Welche Lokalisationen an der Wirbelsäule sind insbesondere durch Stürze (bzw. osteoporotisch) bedingt?

Bei dieser Fragestellung wird auf dieselbe Datenbasis wie bei 1.) zurückgegriffen. Aufgrund der moderaten Fallzahl dient diese Fragestellung zur Hypothesengenerierung. Die Analysen sind ebenfalls deskriptiver Natur (Häufigkeiten), stratifiziert für die Frakturlokalisationen an der Wirbelsäule.

3. Welche biomechanischen Sturzmechanismen tragen zu einer Wirbelkörper- bzw. Beckenfraktur bei?

Hierfür wird im Interview versucht, den Sturzmechanismus (initiale Sturzrichtung; Richtung und Lokalisation des primären Aufpralls) möglichst exakt zu rekonstruieren. Die Analysen sind deskriptiver Natur (Häufigkeiten). Geplant ist ein Vergleich mit biomechanischen Daten zu Sturzmechanismen bei Hüftfraktur. Hierfür stehen zum einen (noch nicht publizierte) Videodaten aus Kanada zur Verfügung. Zum anderen werden diese Informationen in einer durch die EU finanzierten Studie ab 2021 erhoben. Das Robert-Bosch-Krankenhaus stellt eines der Studienzentren dar.

4. Welche Parameter tragen positiv und negativ zur Funktionalität, Mobilität, Beeinträchtigung bzw. Teilhabe bei Patientinnen und Patienten mit Wirbelkörper- bzw. Beckenfraktur bei?

Es handelt sich hierbei um einen klassischen prospektiven Ansatz einer Kohortenstudie, bei dem Prädiktoren für relevante Endpunkte wie Funktionalität (z.B. Gehgeschwindigkeit), Mobilität (z.B. kumulative sensor-basierte Gehdauer), Beeinträchtigung (z.B. Limitierung bei den Aktivitäten des täglichen Lebens) und Teilhabe (z.B. soziale Outdoor-Aktivität) untersucht werden. Zur Analyse kommen multivariate Verfahren (z.B. lineare oder logistische Regression) zur Anwendung.

5. Entwicklung eines Alters-Trauma-Registers für die Frakturentitäten Wirbelkörper- und Beckenfraktur

Der Studienansatz wird genutzt, um Aussagekraft und Machbarkeit von Variablen eines Alters-Trauma-Registers für die Frakturentitäten Wirbelkörper- und Beckenfraktur zu testen. Hierbei orientiert sich das Register an dem schon bestehenden AltersTraumaRegister DGU® für Hüftfrakturen, modifiziert dieses aber um spezifische Aspekte von Wirbelkörper- und Beckenfrakturen.

6. Zudem ist der Aufbau einer Datenbank mit Biomaterialien geplant. Die phänotypisch gut charakterisierte Kohorte soll dazu genutzt werden, weitere Fragen im Bereich der Alterstraumatologie und Altersmedizin zu nutzen. Hierfür ist die Asservierung von Biomaterialien vorgesehen.

10. Risiken und Nebenwirkungen

Alle Messinstrumente zur Erhebung der oben beschriebenen Daten wurden bereits in anderen Studien in vergleichbaren geriatrischen Kollektiven (z.B. nach Hüftfraktur) eingesetzt. Dabei zeigte sich, dass daraus für die Patientinnen und Patienten keine Risiken und/oder Nebenwirkungen zu erwarten sind.
Die in der Studie eingesetzten Geräte sind bezüglich ihrer Sicherheit geprüft (Anlagen). Sie werden der zugelassenen Zweckbestimmung entsprechend eingesetzt und nur von Personen betrieben, angewandt und in Stand gehalten, die dafür die erforderliche Ausbildung, Kenntnis und Erfahrung besitzen.

Mögliche Risiken und Komplikationen der Blutentnahme

Bei der zusätzlichen Entnahme von 2 x 9 ml aus dem venösen System ist auch bei alten Pateint*innen mit keiner nennenswerten hämodynamischen Belastung zu rechnen.
Als Folge der Blutentnahme sind Thrombosen, Fehlpunktionen, die irrtümliche Punktion einer Arterie oder eines Nerven oder Infektionen in sehr seltenen Fällen möglich. Eine Nervschädigung je nach betroffenem Nerv kann ein weites Spektrum möglicher Folgen von einer vorübergehenden Schmerzempfindung, kurzfristigen Lähmung, Taubheitsgefühl bis hin zu chronischen, unbeherrschbaren Schmerzen oder andauernder Lähmung nach sich ziehen.
In seltenen Fällen kann es zu einer lokalen Venenreizung, einer oberflächlichen Venenentzündung oder zur Bildung eines lokalen Blutergusses (Hämatom) kommen, das sich nach wenigen Tagen in der Regel ohne weitere therapeutische Maßnahmen zurückbildet.

Bezüglich einer Nutzen-/Risikobewertung entsteht für die Teilnehmer*innen kein unmittelbarer Nutzen aus der Teilnahme an der Datenerhebung. Allerdings werden auffällige Untersuchungsergebnisse nach Rücksprache und Einwilligung der Teilnehmer*innen an den Hausarzt weitergegeben. In Anbetracht des möglichen gesellschaftlichen Nutzens mit der zukünftigen Möglichkeit zur Prädiktion von unerwünschten Ereignissen und der Möglichkeit zur Intervention (z.B. bei Sturzgefahr) und einem sehr geringen Risiko für die Teilnehmerinnen und Teilnehmer erscheint uns die Durchführung der Datenerhebung gerechtfertigt.

11. Abbruchkriterien

Die Datenerhebung kann bei Auftreten von Komplikationen oder auf ausdrücklichen Wunsch der Teilnehmer hin abgebrochen werden. Solche Komplikationen können in jeglicher Form und Ausmaß von Unwohlsein oder Angst auftreten, wenn dies durch die Untersuchung oder aus anderen Gründen zur Zeit der Untersuchung auftritt. Ebenso können Teilnehmer*innen jederzeit ohne Angaben von Gründen ihr/sein Einverständnis zurückziehen. Bei einem Rücktritt von der Studie können die Teilnehmer*innen selbst entscheiden, ob bereits vorhandene Daten weiterverwendet werden dürfen oder gelöscht werden müssen.

Beim Auftreten von nicht vorhergesehenen Ereignissen, die studienbedingt zum Nachteil der Teilnehmer*innen beitragen, entscheidet die Studienleitung (i.e. medizinisch Verantwortlicher) über den Abbruch der Studie.

Die Studienleitung behält sich ebenso das Recht vor, bei einzelnen Teilnehmer*innen die Untersuchung vorzeitig zu beenden, wenn der Gesundheitszustand der Probandin/des Probanden dies erfordert. Bis zu diesem Zeitpunkt erhobene Messwerte können jedoch zur Auswertung herangezogen werden.

12. Datenschutz

Alle Projektmitarbeiter unterliegen der Schweigepflicht. Informationsbogen und Einwilligungserklärung sind an die geltende Datenschutzgrundverordnung angepasst. Die in der Studie erhobenen Patientendaten werden von den Verantwortlichen vertraulich behandelt und vor unberechtigtem Zugriff geschützt. Als verantwortliche Person für die Datenerhebung wird in dieser Studie am RBK sowie für die Datenverarbeitung im Rahmen der Biodatenbank Herr Prof. Rapp benannt. Als verantwortliche Person für die Datenerhebung an dem jeweiligen Studienzentrum wird der Projektleiter des Studienzentrums benannt.

In einer gesonderten Patienten-Aufklärung werden die Patienten darüber informiert und werden gebeten, Biomaterialien für zusätzliche wissenschaftliche Untersuchungen im Rahmen einer Biodatenbank zur Verfügung zu stellen. Desweiterem werden die Studienteilnehmer informiert, dass mit der Überlassung der Biomaterialien an die Biodatenbank diese das Eigentum der Robert-Bosch-Krankenhaus GmbH und der Robert Bosch Gesellschaft für medizinische Forschung mbH werden.

Die Einwilligung für Biodatenbank ist freiwillig und beeinflusst nicht die Teilnahme an der o.g. Studie.

Die Dokumentation der erhobenen Daten und deren Archivierung erfolgt pseudonymisiert in einem geschützten Studienfile, zu dem nur befugte Mitarbeiter*innen einschließlich auf das Berufs- und Datengeheimnis verpflichteter Doktorand*innen Zutritt haben.

Alle Daten werden pseudonymisiert und im Robert-Bosch-Krankenhaus gespeichert. Hierzu wird dem Datensatz einer Person eine ID-Nummer zugeteilt. Eine Liste, die ID-Nummern und Namen verbindet, steht für mögliche Rücktritte und Datenlöschungen nur der Studienleitung und der Projektkoordination des Prüfzentrums zur Verfügung. Die schriftlichen Aufzeichnungen werden in einem geschlossenen Raum aufbewahrt und nach 10 Jahren vernichtet. Die Daten werden in anonymer Form nach Beendigung der Studie bei Bedarf zur wissenschaftlichen Auswertung öffentlich zugänglich gemacht. Persönliche Daten, wie z.B. Namen und Kontaktinformationen verbleiben im Studienzentrum und werden nicht weitergegeben. Alle Teilnehmer*innen werden über die Handhabung mit den Daten und deren Auswertungen informiert.

Im Falle eines Widerrufs der Einwilligung durch den Patienten kann der Patient freiwillig entscheiden, ob bis dahin bereits erhobene Daten weiterhin gespeichert bleiben und verwendet werden können. Sollte durch den Patienten eine ausdrückliche Löschung der Daten verlang werden, müssen die Daten komplett gelöscht werden.

Für den Fall, dass eine ausdrückliche Vernichtung des Probenmaterials von Patientenseite auch nach erfolgter Einwilligung gewünscht wird, wird anhand der geführten Identifikationsliste dem betroffenen Patienten seine Probe zugeordnet und vernichtet.

Alle erhobenen persönlichen und klinischen Daten sowie alle gewonnenen Biomaterialien werden für einen unbefristeten Zeitraum aufbewahrt. Spätestens 10 Jahre nach Erteilung der Einwilligung zur Teilnahme an der Studie anonymisiert. Ein Widerruf der Einwilligung sowie Daten- und Probenvernichtung nach erfolgter Anonymisierung ist nicht möglich, worauf die Studienteilnehmer*innen hingewiesen werden.

Die im Rahmen der Studie erhobenen Daten können auch für künftige Forschungsvorhaben der Klinik bzw. des Instituts genutzt und weiterverarbeitet werden.

Die Weitergabe von Daten im Rahmen der Studienauswertung und sonstiger Analysen der Ergebnisse an Dritte (Universitäten, Forschungsinstitute und forschende Unternehmen zu Zwecken medizinischer Forschung) erfolgt pseudonymisiert und nur im notwendigen Umfang. Dies schließt unter Umständen auch die Weitergabe für Forschungsprojekte im Ausland bzw. an Kooperationspartner außerhalb des Europäischen Wirtschaftsraumes, d.h. in Länder mit geringerem Datenschutzniveau (z.B. die USA) ein. In solchen Fällen wird, soweit rechtlich möglich, vereinbart, dass die Kooperationspartner verpflichtet sind, das EU-Datenschutz-Niveau einzuhalten. Dennoch ist nicht in jedem Fall auszuschließen, dass alle Bedingungen exakt eingehalten werden und ggf. unabhängige Aufsichtsbehörden nicht zur Verfügung stehen, die den Teilnehmer bei der Wahrnehmung seiner Betroffenenrechte unterstützen können. Die Patienten werden darüber aufgeklärt.

Für die Weitergabe von Daten an externe Forscher erfolgt eine doppelte Pseudonymisierung.

Die Forschungsergebnisse aus der Studie werden in anonymisierter Form in Fachzeitschriften oder in wissenschaftlichen Datenbanken veröffentlicht.
Die Studienteilnehmer*innen werden in der Patienteninformation darauf hingewiesen, dass sie jederzeit Auskunft über ihre gespeicherten Daten (inkl. Überlassung einer kostenlosen Kopie) verlangen können und das Recht haben, fehlerhafte Daten berichtigen zu lassen. Die Studienteilnehmer*innen können auch jederzeit verlangen, dass ihre Daten gelöscht oder anonymisiert werden und sich bei Beschwerden an den Datenschutzbeauftragten des Robert-Bosch-Krankenhauses und/oder an den Bundesdatenschutzbeauftragten bzw. den Landesdatenschutzbeauftragten wenden.
Rechtsgrundlage für die Verarbeitung Ihrer Daten sind Art. 6, 7, 9, 89 der Datenschutz-Grundverordnung in Verbindung mit §§ 22, 27, 29, 32, 33, 34, 35, 36, 38 in der Fassung des Datenschutz- Anpassungs- und Umsetzungsgesetzes EU v. 30. Juni 2017, BGBl. I, S. 2097 ff.
Hinweis für Prüfstellen, die dem LDSG unterliegen:
Rechtsgrundlage für die Verarbeitung Ihrer Daten sind Art. 6, 7, 9, 89 der Datenschutz-Grundverordnung in Verbindung mit §§ 4, 5, 6, 8, 9, 12, 13 des Landesdatenschutzgesetzes Baden-Württemberg in der ab 25. Mai 2018 geltenden Fassung.

13. Ethische Belange

Die Studie wird nach Maßgaben der „Good Clinical Practice" und in Übereinstimmung mit den Grundsätzen der Deklaration von Helsinki 1964 und allen nachfolgenden Überarbeitungen durchgeführt. Alle Teilnehmer*innen müssen eine schriftliche Einverständniserklärung unterzeichnen. Eine berufsrechtliche Beratung der Studie durch die zuständige Ethik-Kommission wird durchgeführt. Bevor die Ethik-Kommission ihre positive Einschätzung der Abläufe dargestellt hat, werden keine Personen in die Studie aufgenommen. Die Projektleitung wird die Empfehlungen der Ethik-Kommission berücksichtigen.

14. Aufklärung der Studienteilnehmer

Alle möglichen Teilnehmer*innen werden im RBK angesprochen. Sie werden nach Interessensbekundung über die Studie und die Möglichkeit der Teilnahme mündlich und schriftlich informiert. Ein Exemplar der Studieninformation und eine Einverständniserklärung und Datenschutzerklärung wird überreicht und verbleibt beim Interessenten. Alle Teilnehmer*innen werden informiert, dass eine Teilnahme oder Nicht-Teilnahme keinen Einfluss auf die Behandlung im RBK hat. Sie werden auch explizit darüber informiert, dass RBK-Routinedaten in die Studie einfließen. Vor der ersten Follow-Up-Untersuchung muss die Einverständnis- und Datenschutzerklärung von den Teilnehmer*innen unterschrieben sein.

Vor der Unterschrift und vor der ersten Messung erfolgt dann eine zusätzliche Aufklärung, bei der noch mögliche letzte Fragen der Teilnehmer*innen beantwortet werden.
Es können auch Teile der Studie, wie die Nutzung von Biomaterialien abgelehnt werden, ohne dass dadurch ein Ausschluss aus den restlichen Anteilen der Studie folgt.

15. Anlagen

- Information über die Studie für Interessierte
- Einwilligungserklärung
- Patientinnen-Information und Einwilligungserklärung in die Verwendung von Biomaterialien und zugehörigen Daten in Biodatenbank
- Datenerhebungsbögen
- Beschreibung/Sicherheitsbeurkundungen der eingesetzten Methoden

16. Unterschriften

Stuttgart, 23.02.2021 Prof. Dr. Clemens Becker
Chefarzt der Klinik für Geriatrische Rehabilitation,
Robert-Bosch-Krankenhaus, Stuttgart

Stuttgart, 23.02.2021 Prof. Dr. med. Bernd Kinner
Chefarzt der Abteilung Orthopädie und Unfallchirurgie,
Robert-Bosch-Krankenhaus Stuttgart

17. Literatur

Andrich, S., Haastert, B., Neuhaus, E., Neidert, K., Arend, W., Ohmann, C., Grebe, J., Vogt, A., Jungbluth, P., Rösler, G., Windolf, J., Icks, A., 2015. Epidemiology of Pelvic Fractures in Germany: Considerably High Incidence Rates among Older People. PloS One 10, e0139078. https://doi.org/10.1371/journal.pone.0139078

Autier, P., Haentjens, P., Bentin, J., Baillon, J.M., Grivegnée, A.R., Closon, M.C., Boonen, S., 2000. Costs induced by hip fractures: a prospective controlled study in Belgium. Belgian Hip Fracture Study Group. Osteoporos. Int. J. Establ. Result Coop. Eur. Found. Osteoporos. Natl. Osteoporos. Found. USA 11, 373–380. https://doi.org/10.1007/s001980070102

Baker, P.S., Bodner, E.V., Allman, R.M., 2003. Measuring life-space mobility in community-dwelling older adults. J. Am. Geriatr. Soc. 51, 1610–1614. https://doi.org/10.1046/j.1532-5415.2003.51512.x

Benzinger, P., Riem, S., Bauer, J., Jaensch, A., Becker, C., Büchele, G., Rapp, K., 2019. Risk of institutionalization following fragility fractures in older people. Osteoporos. Int. J. Establ. Result Coop. Eur. Found. Osteoporos. Natl. Osteoporos. Found. USA 30, 1363–1370. https://doi.org/10.1007/s00198-019-04922-x

Charlson, M.E., Pompei, P., Ales, K.L., MacKenzie, C.R., 1987. A new method of classifying prognostic comorbidity in longitudinal studies: development and validation. J. Chronic Dis. 40, 373–383. https://doi.org/10.1016/0021-9681(87)90171-8

Cumming, R.G., Klineberg, R., Katelaris, A., 1996. Cohort study of risk of institutionalisation after hip fracture. Aust. N. Z. J. Public Health 20, 579–582. https://doi.org/10.1111/j.1467-842x.1996.tb01069.x

Cummings, S.R., Melton, L.J., 2002. Epidemiology and outcomes of osteoporotic fractures. Lancet Lond. Engl. 359, 1761–1767. https://doi.org/10.1016/S0140-6736(02)08657-9

de Morton, N.A., Davidson, M., Keating, J.L., 2008. The de Morton Mobility Index (DEMMI): an essential health index for an ageing world. Health Qual. Life Outcomes 6, 63. https://doi.org/10.1186/1477-7525-6-63

Delgard, O.S., 1996. Community health profile: A tool for psychiatric prevention, in: Trend, D.R., Reed, C.A. (Eds.), Promotion of Mental Health. Aldershot, Avebury, pp. 395–402.

Dyer, S.M., Crotty, M., Fairhall, N., Magaziner, J., Beaupre, L.A., Cameron, I.D., Sherrington, C., Fragility Fracture Network (FFN) Rehabilitation Research Special Interest Group, 2016. A critical review of the long-term disability outcomes following hip fracture. BMC Geriatr. 16, 158. https://doi.org/10.1186/s12877-016-0332-0

Guralnik, J.M., Simonsick, E.M., Ferrucci, L., Glynn, R.J., Berkman, L.F., Blazer, D.G., Scherr, P.A., Wallace, R.B., 1994. A short physical performance battery assessing lower extremity function: association with self-reported disability and prediction of mortality and nursing home admission. J. Gerontol. 49, M85-94. https://doi.org/10.1093/geronj/49.2.m85

Heidenblut, S., Zank, S., 2010. [Development of a new screening instrument for geriatric depression. The depression in old age scale (DIA-S)]. Z. Gerontol. Geriatr. 43, 170–176. https://doi.org/10.1007/s00391-009-0067-z

Hernlund, E., Svedbom, A., Ivergård, M., Compston, J., Cooper, C., Stenmark, J., McCloskey, E.V., Jönsson, B., Kanis, J.A., 2013. Osteoporosis in the European Union: medical management, epidemiology and economic burden. A report prepared in collaboration with the International Osteoporosis Foundation (IOF) and the European Federation of Pharmaceutical Industry Associations (EFPIA). Arch. Osteoporos. 8, 136. https://doi.org/10.1007/s11657-013-0136-1

Höch, A., Pieroh, P., Gras, F., Hohmann, T., Märdian, S., Holmenschlager, F., Keil, H., Palm, H.-G., Herath, S.C., Josten, C., Schmal, H., Stuby, F.M., Pelvic Injury Register of the German Trauma Society, 2019. Age and “general health”-beside fracture classification-affect the therapeutic decision for geriatric pelvic ring fractures: a

German pelvic injury register study. Int. Orthop. https://doi.org/10.1007/s00264-019-04326-w

Icks, A., Arend, W., Becker, C., Rapp, K., Jungbluth, P., Haastert, B., 2013. Incidence of hip fractures in Germany, 1995-2010. Arch. Osteoporos. 8, 140. https://doi.org/10.1007/s11657-013-0140-5

Kanis, J.A., Johnell, O., Oden, A., Jonsson, B., De Laet, C., Dawson, A., 2000. Risk of hip fracture according to the World Health Organization criteria for osteopenia and osteoporosis. Bone 27, 585–590.

Katzman, R., Brown, T., Fuld, P., Peck, A., Schechter, R., Schimmel, H., 1983. Validation of a short Orientation-Memory-Concentration Test of cognitive impairment. Am. J. Psychiatry 140, 734–739. https://doi.org/10.1176/ajp.140.6.734

Magaziner, J., Fredman, L., Hawkes, W., Hebel, J.R., Zimmerman, S., Orwig, D.L., Wehren, L., 2003. Changes in functional status attributable to hip fracture: a comparison of hip fracture patients to community-dwelling aged. Am. J. Epidemiol. 157, 1023–1031. https://doi.org/10.1093/aje/kwg081

Mahoney, F.I., Barthel, D.W., 1965. FUNCTIONAL EVALUATION: THE BARTHEL INDEX. Md. State Med. J. 14, 61–65.

Middleton, R.G., Shabani, F., Uzoigwe, C.E., Shoaib, A., Moqsith, M., Venkatesan, M., 2012. FRAX and the assessment of the risk of developing a fragility fracture. J. Bone Joint Surg. Br. 94, 1313–1320. https://doi.org/10.1302/0301-620X.94B10.28889

Oberkircher, L., Ruchholtz, S., Rommens, P.M., Hofmann, A., Bücking, B., Krüger, A., 2018. Osteoporotic Pelvic Fractures. Dtsch. Arzteblatt Int. 115, 70–80. https://doi.org/10.3238/arztebl.2018.0070

Ong, T., Kantachuvesiri, P., Sahota, O., Gladman, J.R.F., 2018. Characteristics and outcomes of hospitalised patients with vertebral fragility fractures: a systematic review. Age Ageing 47, 17–25. https://doi.org/10.1093/ageing/afx079

Orwig, D., Hochberg, M.C., Gruber-Baldini, A.L., Resnick, B., Miller, R.R., Hicks, G.E., Cappola, A.R., Shardell, M., Sterling, R., Hebel, J.R., Johnson, R., Magaziner, J., 2018. Examining Differences in Recovery Outcomes between Male and Female Hip Fracture Patients: Design and Baseline Results of a Prospective Cohort Study from the Baltimore Hip Studies. J. Frailty Aging 7, 162–169. https://doi.org/10.14283/jfa.2018.15

Parker, M.J., Palmer, C.R., 1993. A new mobility score for predicting mortality after hip fracture. J. Bone Joint Surg. Br. 75, 797–798.

Rabin, R., de Charro, F., 2001. EQ-5D: a measure of health status from the EuroQol Group. Ann. Med. 33, 337–343. https://doi.org/10.3109/07853890109002087

Rapp, K., Büchele, G., Dreinhöfer, K., Bücking, B., Becker, C., Benzinger, P., 2019. Epidemiology of hip fractures : Systematic literature review of German data and an overview of the international literature. Z. Gerontol. Geriatr. 52, 10–16. https://doi.org/10.1007/s00391-018-1382-z

Ross, P.D., 1997. Clinical consequences of vertebral fractures. Am. J. Med. 103, 30S-42S; discussion 42S-43S. https://doi.org/10.1016/s0002-9343(97)90025-5

Schulz, C., Büchele, G., Rehm, M., Rothenbacher, D., Roigk, P., Rapp, K., Günster, C., König, H.-H., Reber, K., 2019. Patient Characteristics as Indicator for Care Dependence after Hip Fracture: A Retrospective Cohort Study Using Health Insurance Claims Data From Germany. J. Am. Med. Dir. Assoc. 20, 451-455.e3. https://doi.org/10.1016/j.jamda.2018.09.029

Tinetti, M.E., Ginter, S.F., 1990. The nursing home life-space diameter. A measure of extent and frequency of mobility among nursing home residents. J. Am. Geriatr. Soc. 38, 1311–1315. https://doi.org/10.1111/j.1532-5415.1990.tb03453.x

van Schoor, N.M., Knol, D.L., Glas, C. a. W., Ostelo, R.W.J.G., Leplège, A., Cooper, C., Johnell, O., Lips, P., 2006. Development of the Qualeffo-31, an osteoporosis-specific quality-of-life questionnaire. Osteoporos. Int. J. Establ. Result Coop. Eur. Found. Osteoporos. Natl. Osteoporos. Found. USA 17, 543–551. https://doi.org/10.1007/s00198-005-0024-7

Wong, R.M.Y., Wong, H., Zhang, N., Chow, S.K.H., Chau, W.W., Wang, J., Chim, Y.N., Leung, K.S., Cheung, W.H., 2019. The relationship between sarcopenia and fragility fracture-a systematic review. Osteoporos. Int. J. Establ. Result Coop. Eur. Found. Osteoporos. Natl. Osteoporos. Found. USA 30, 541–553. https://doi.org/10.1007/s00198-018-04828-0

Yardley, L., Beyer, N., Hauer, K., Kempen, G., Piot-Ziegler, C., Todd, C., 2005. Development and initial validation of the Falls Efficacy Scale-International (FES-I). Age Ageing 34, 614–619. https://doi.org/10.1093/ageing/afi196
